# Adaptive multimodal swimming gaits in a reconfigurable modular soft robotic fish

**DOI:** 10.1126/sciadv.aea1299

**Published:** 2026-01-02

**Authors:** Bo Wang, Lei Li, Mengfan Xu, Nannan Hu, Wenzhuo Gao, Jie Zhang, Bo Yin, Zhanhua Xin, Junzhi Yu

**Affiliations:** ^1^ State Key Laboratory for Turbulence and Complex Systems, School of Advanced Manufacturing and Robotics, Peking University, Beijing 100871, China.; ^2^ Institute of Ocean Research, Peking University, Beijing 100871, China.; ^3^ Institute of Mechanics, Chinese Academy of Sciences, Beijing 100190, China.; ^4^ School of Mechanics and Aerospace Engineering, Dalian University of Technology, Dalian 116024, China.

## Abstract

Fish swim with four main gaits—anguilliform, subcarangiform, carangiform, and thunniform—produced by waves along varying portions of the body. However, how muscle activation length influences swimming performance remains poorly understood. We present a reconfigurable robotic fish that replicates all four gaits in a single platform by rapidly tuning its body stiffness. Vacuum-driven layer jamming muscles in four tensegrity joints enable quick (≤1 s) stiffness modulation (stiffness ratio of 46.6) and gait switching. In thunniform gait, the robot reaches 1.24 body lengths per second, whereas in anguilliform gait, it achieves agile maneuvering with a turning radius of 0.26 body lengths. Fluid simulations show that the thunniform gait generates stronger vortices and 142% more thrust compared with anguilliform motion at 5 Hz, explaining its high-speed performance. The robot dynamically adapts gaits during locomotion—using thunniform for fast traversal and anguilliform for obstacle negotiation—demonstrating environmental adaptability. This work advances understanding of aquatic multimodal locomotion.

## Teaser

Bioinspired robotic fish with programmable stiffness mimics four gaits, enabling adaptive swimming in varied environments.

## INTRODUCTION

Body/caudal fin (BCF) fish exhibit remarkable swimming versatility in response to diverse ecological demands (*1*–*3*), ranging from rapid bursts for predator evasion and prey capture (*4*, *5*) to slow, sustained cruising during long-distance migrations (*6*–*8*). This versatility arises from their ability to recruit different portions of the body into wave motions, resulting in four classical swimming gaits: anguilliform, subcarangiform, carangiform, and thunniform (*9*, *10*). Swimming speed increases along this continuum, from the low-speed, eel-like anguilliform motion [<3 body lengths per second (BL/s)] (*11*–*15*) to the high-speed, tuna-like thunniform mode (up to 20 BL/s) (*3*, *16*–*19*), with trout- and mackerel-like forms in between. Previous studies have investigated how fin shape (*20*), skin morphology (*21*–*23*), muscle mechanics (*24*–*26*), and body stiffness (*27*–*29*) affect swimming performance. However, the role of the axial body length engaged in generating undulatory waves remains a potentially crucial factor that is still poorly understood.

In detail, the four BCF swimming modes differ markedly in how much of the body undulates. In anguilliform swimming, more than 80% of the body length participates in undulation, whereas subcarangiform swimmers engage roughly 50 to 70% of the body. Carangiform swimmers limit undulation to the posterior one-third (about 30 to 50%), and thunniform swimmers confine motion to the caudal peduncle (<20% BL), with the anterior body nearly rigid (*14*, *30*–*32*). These kinematic patterns are driven by the sequential activation of discrete axial muscle groups: Activation begins near the head and propagates toward the tail through neural delays to generate thrust (*25*, *33*, *34*). Eel-like swimmers, activating almost the entire trunk each cycle, produce short body-wave wavelengths (<1 BL) (*14*, *33*–*37*), whereas tuna-like swimmers confine activation to the posterior region, producing long, rapidly propagating wavelengths (>1 BL) (*32*, *33*, *35*, *38*). Such neuromuscular distinctions underlie the transition across swimming modes (*34*).

Although it is well accepted that body undulations arise from sequential muscle activation along the fish trunk, the extent to which the length of active musculature influences swimming performance remains unclear. In vivo, electromyography is the primary tool for measuring muscle activation patterns (*33*–*35*, *39*–*41*), but it requires invasive electrodes and samples only discrete body sites. This limitation makes comprehensive, noninvasive mapping of full-body muscle recruitment infeasible (*42*), constraining our understanding of how different axial activation strategies affect locomotion.

To address this gap, robophysical models—bioinspired robots designed to test biological hypotheses—offer a promising approach (*43*, *44*). Diverse robotic fish have been built to emulate each of the four BCF swimming gaits. Anguilliform-inspired robots use flexible or segmented bodies to replicate full-body undulation, resulting in high maneuverability in confined spaces at low speeds (~0.1 to 1.0 BL/s) (*45*–*49*). Carangiform and subcarangiform robots typically feature a rigid anterior body and localized bending to the tail region, allowing them to achieve higher swimming speeds than their anguilliform counterparts (*50*–*55*). Specialized thunniform robots, modeled after tunas, combine a rigid front body with a flexible caudal peduncle, enabling high-frequency tail oscillations (up to 15 Hz) that produce exceptional thrust-to-power ratios and sustained speeds above 4.6 BL/s through efficient propulsion (*56*–*59*). However, these existing robots are generally constrained to a single fixed mode; each design’s unique morphology, propulsion mechanism, and hydrodynamic interaction make direct comparisons across modes difficult, thereby raising development costs and time. These challenges motivate the need for a modular, reconfigurable robotic platform that can systematically switch among multiple locomotion modes.

Truly multimodal robotic fish remain scarce. For example, Wang *et al.* developed a soft robotic fish with four dielectric elastomer actuators to switch modes via amplitude-phase modulation (*60*). However, their design uses two symmetric actuator pairs, effectively dividing the body into just anterior and posterior segments. All actuators activate simultaneously, preventing selective control over the axial length involved in propulsion. These limitations hinder the faithful replication of natural fishes’ distributed muscle-activation patterns and impede a systematic study of how different activation strategies, body-wave dynamics, and swimming performances are related.

**Fig. 1.**
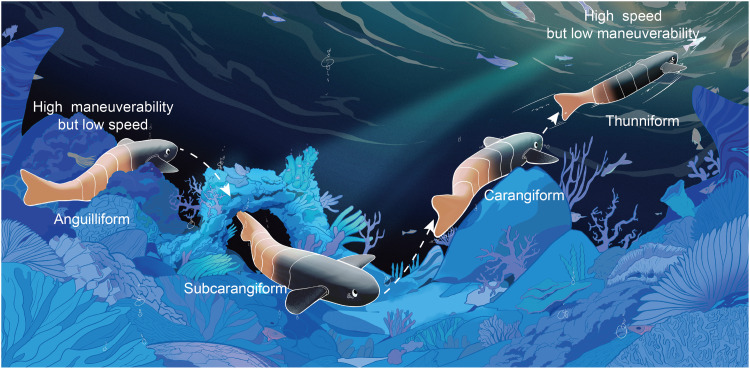
A multimodal robotic fish capable of replicating four distinct swimming modes: anguilliform, subcarangiform, carangiform, and thunniform. This reconfigurable design enables adaptive locomotion across varied environments, leveraging anguilliform undulation for high maneuverability in cluttered settings and switch. The illustration was created using Procreate software.

Here, inspired by the discrete activation and stiffness-locking mechanisms of natural fish musculature (*20*, *33*, *39*, *61*), we develop a multimodal soft robotic fish that integrates the in situ reconfigurability of tensegrity structures with rapid stiffness modulation via layer jamming technology. Our design features a tail with four sequential joints that can be selectively activated. By choosing which joints to lock, from head to tail, we can replicate anguilliform, subcarangiform, carangiform, or thunniform swimming gaits (Fig. 1). Using this bioinspired, reconfigurable platform, we systematically investigate how different axial muscle-activation patterns affect swimming performance. We also perform computational fluid dynamics (CFD) simulations across all four gaits to elucidate the underlying hydrodynamics. Last, we demonstrate the robot’s adaptability through field tests, where it seamlessly switches among locomotion modes in response to environmental demands, highlighting its potential as a task-adaptive robotic system for complex aquatic missions and as a physical model for studying fish swimming strategies.

## RESULTS

### Design and fabrication of a multimodal soft robotic fish

**Fig. 2.**
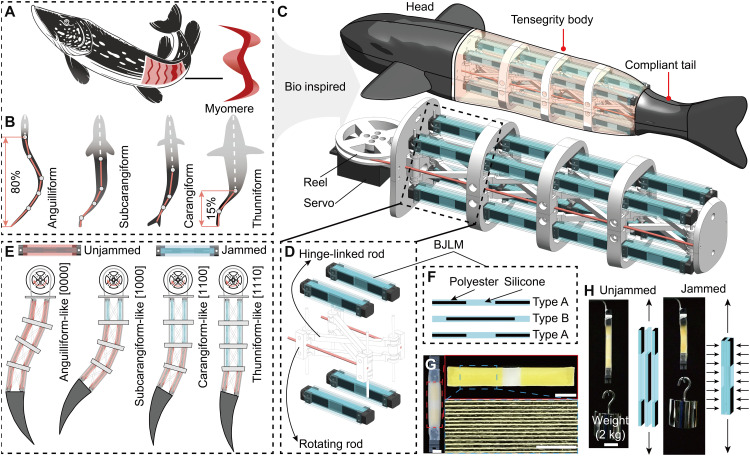
Design of the multimodal soft robotic fish. (**A**) Natural fish myotomes are arranged as paired, W-shaped segments along the body sides; right: schematic of a single myotome unit. (**B**) Four swimming modes: anguilliform (six segments; red lines indicate active body undulation, white dotted line denotes rigidity), subcarangiform (five segments, posterior undulation), carangiform (four segments, caudal-peduncle undulation), and thunniform (three segments, minimal tail undulation). (**C**) Prototype structure: a rigid head module, a tensegrity-based multijoint body, and a flexible caudal fin. (**D**) One tensegrity joint consists of four rotating rods, four hinge-linked rods, and four bioinspired jamming layer muscles (BJLMs) arranged in two opposing pairs. (**E**) Illustration of the four swimming modes of the robotic fish. Locking joints sequentially from head to tail generate four modalities, each using a specific number of active segments. (**F**) Schematic of segmented elastic layers (SELs). Each SEL features alternating inextensible polyester thread (black) and elastic silicone (blue) segments. (**G**) A BJLM with nine SELs (5-cm length) at atmospheric pressure. Scale bar, 1 mm. Close-up images show the microstructure of SELs (top; scale bar, 1 mm) and a polyester-silicone interface (bottom; scale bar, 100 μm), highlighting discontinuous polyester embedding. (**H**) Mechanical response of BJLM to vacuum actuation. At atmospheric pressure, SELs slide freely, enabling high stretchability (61% strain under a 2-kg load). Applying a –60-kPa vacuum compresses the layers, increasing interlayer friction through overlapping polyester sections and restricting strain to 4% (movie S1 and fig. S5). Scale bar, 2 cm.

Fish body musculature is organized in repeating W-shaped segments on both sides, which are segmentally arranged into blocks called myomeres, separated by myosepts (*62*) (Fig. 2A). This modular muscle activation enables diverse swimming gaits, where the “muscle recruitment length”—the body portion engaged in undulatory motion—critically influences swimming performance (Fig. 2B). Fish bodies can thus be conceptually modeled as a series of pseudojoints: Anguilliform swimmers typically engage six segments, subcarangiform five, carangiform four, and thunniform three (*63*, *64*). In Fig. 2B, white dashed lines denote relatively rigid body regions, whereas red solid lines indicate compliant segments involved in active bending.

Inspired by these natural templates, we designed a soft robotic fish with a modular tensegrity body that reproduces segmented muscle recruitment and enables seamless switching among four distinct swimming modes (Fig. 2C). The robotic fish consists of three main parts: a rigid three-dimensionally (3D) printed head (housing the control electronics), a reconfigurable tensegrity midbody, and a compliant caudal fin. The tensegrity body is composed of four identical tensegrity joints. Each joint consists of two square, ring-shaped plates connected by four rotating rods and four hinge-linked rods. Four bioinspired jamming layer muscles (BJLMs) brace each joint; these elements are arranged as two antagonistic pairs (Fig. 2D). Adjusting the vacuum pressure in each BJLM tunes its stiffness, enabling rapid joint locking or release. Vacuum is regulated by an onboard pump in the head (see fig. S1 and text S1 for details).

Cables driven by a servo in the head actuate the four tensegrity joints along the body. Each joint can be switched between a compliant (0, unjammed) state and a rigid (1, jammed) state via its vacuum-actuated BJLMs. We defined four swimming modes by the pattern of joint activation from head to tail (Fig. 2E): [0000] anguilliform (all joints compliant), [1000] subcarangiform (first joint rigid), [1100] carangiform (first two rigid), and [1110] thunniform (first three rigid). The compliant caudal fin, made of soft material reinforced with a carbon fiber plate, is attached to the last joint (fig. S2 and text S2).

Each BJLM is constructed from segmented elastic layers (SELs), which are sheets of alternating inextensible polyester thread and compliant silicone (Fig. 2F). We developed two types of SEL (type A and type B), each embedding a unique discontinuous pattern of polyester in a silicone sheet. To assemble a BJLM, several type A and type B SEL strips are aligned and bonded at their ends. They are then placed inside a sealed silicone envelope and connected to a vacuum source (Fig. 2G). Full fabrication details are provided in Materials and Methods and the Supplementary Materials (figs. S3 and S4 and text S3). Within each type of SEL, the length of polyester content is over 50% of the length, so adjacent layers of the two types have overlapping polyester segments. In the unjammed state (no vacuum), the layers slide freely without contact, so the BJLM’s stiffness is governed by the stiffness of silicone segments in each layer (*k*_s_), sections with embedded polyester thread (*k*_p_), and the surrounding membrane (*k*_m_) (Fig. 2H, figs. S5 and S6, and text S4). This segmented design makes the BJLM (nine SELs) highly stretchable, as it elongates by 61% under a 2-kg load and up to 200% (twice its original length) before failing (fig. S7). When a vacuum is applied, the layers compress, and friction at the overlapping polyester-polyester (*k*_pp_), polyester-silicone (*k*_ps_), and polyester-membrane (*k*_pm_) interfaces increases dramatically (fig. S5 and text S4). In the jammed state, these interlayer shear forces allow the BJLM to resist extension and restrict tensile strain to 4% at *P* = −60 kPa (Fig. 2H and movie S1).

**Fig. 3.**
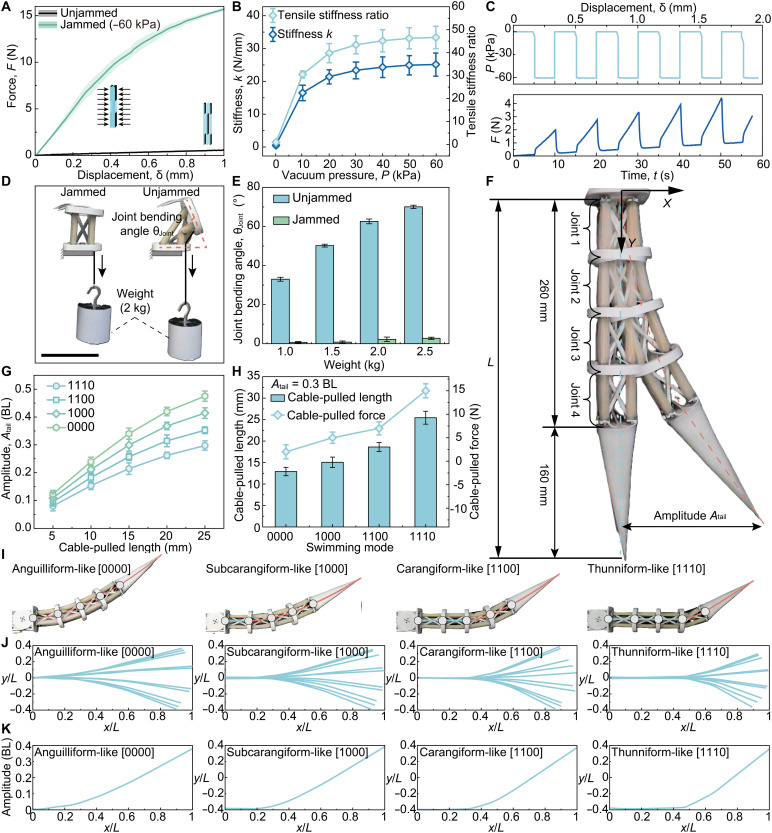
Characterization of the BJLM and multimodal tensegrity body. (**A**) Force-displacement curves of the BJLM (70% polyester, nine SELs) under jammed (−60 kPa) and unjammed (0 kPa). Solid lines: mean response (*n* = 5); shaded areas: ±1 SD. (**B**) Jammed stiffness and stiffness ratio as functions of vacuum pressure (70% polyester, nine SELs, *n* = 5). (**C**) Dynamic stiffness modulation during midtest jamming and unjamming. Time profiles of vacuum pressure and corresponding force response. Solid lines: mean response (*n* = 5); shaded areas: ±1 SD. (**D**) A tensegrity joint holding a 2-kg weight, shown in jammed and unjammed states (movie S2). Scale bar, 10 cm. (**E**) Joint bending angle θ_Joint_ versus load (1 to 2.5 kg) under jammed and unjammed states (*n* = 3). (**F**) Physical prototype with four tensegrity joints. (**G**) Tail amplitude *A*_tail_ versus cable-pulled length across locomotion modes (*n* = 3). (**H**) Required cable-pulled length to achieve a fixed 0.3-BL *A*_tail_ and required cable-pulled force in each mode (*n* = 3). (**I**) Representative configurations of the four gaits (movie S3). (**J**) Midline displacement of entire robot *y*/*L* along the body *x*/*L* for different gaits. (**K**) Along-body amplitude distribution for each gait. All error bars represent ±1 SD.

To finalize the BJLM design, we characterized its mechanical performance under various configurations. Theoretical modeling (fig. S5 and text S4) indicated that the BJLM’s tensile stiffness *k* depends on polyester length fraction (γ), number of layers (*N*), and vacuum pressure (*P*). We performed uniaxial tensile tests while varying γ, *N*, and *P*. The quasistatic tension results showed that the stiffness of BJLM increases with higher γ, more layers *N*, and stronger vacuum (*P* < 0) (figs. S8 to S10). Based on these tests, we chose γ = 70% and *N* = 9 for the final design, which maximized the stiffness ratio to 46.60 ± 4.86 at *P* = −60 kPa (Fig. 3, A and B). This high stiffness modulation is critical for switching locomotion modes, as each tensegrity joint can smoothly alternate between compliant (unjammed) and rigid (jammed) states to mimic muscle recruitment in real fish. The BJLM maintains low bending in orthogonal directions and buckling stiffness, which is also critical for preserving joint flexibility during undulatory motion (fig. S11).

Under cyclic loading test, the BJLM exhibits durable and fully reversible behavior (fig. S12). In the jammed state (*P* = −60 kPa, initial tension of 11.3 N), it retained over 75% of its tensile force after 1000 stretch cycles. This slight loss in force was due to minor interlayer slip, with full recovery upon unjamming. Alternating between jammed and unjammed states for 100 cycles produced negligible changes in the force-displacement response, with consistent stiffness at each target displacement. In addition, the BJLM exhibited rapid switching between states, with a transition time of 0.5 s (Fig. 3C and fig. S13). Overall, the BJLM achieves a large contrast in tensile stiffness while maintaining flexibility and durability. This combination enables the robotic fish to execute rapid, repeatable switches among its swimming modes while remaining reliable. Details of the BJLM performance tests are provided in text S5.

### Multimodal locomotion implementation in robotic fish

To validate mode switching, we performed a clamp-free bending test on a tensegrity joint (Fig. 3D and movie S2). In its unjammed state, the joint bent to an angle θ_Joint_ of 33° ± 1° under a 1-kg load and 70° ± 1° under 2.5 kg. Applying a −60-kPa vacuum to BJLMs reduced the 2.5-kg bending to just 2.8° ± 0.6° (Fig. 3E), demonstrating effective pneumatic control of joint stiffness. This locking action is key for switching swimming modes by locking or unlocking segments. Next, we assembled four such tensegrity joints in series to demonstrate multimodal locomotion (Fig. 3F). Each joint’s state was controlled by solenoid valves, and a cable provided the bending actuation (fig. S1 and text S1). We observed that tail amplitude *A*_tail_ increased with actuation displacement (Fig. 3G). For example, at the same displacement, the anguilliform-like [0000] mode produced tail amplitudes 1.7 times larger than the thunniform-like [1110] mode.

To standardize amplitude to 0.3 BL, we adjusted the actuation displacements for each gait: 12.9 mm for [0000], 15.1 mm for [1000], 18.6 mm for [1100], and 25.4 mm for [1110] (Fig. 3H). At those matched amplitudes, the required cable-pulling force increased markedly with the joint locking number with values of 2.1 ± 1.5 N [0000], 5.2 ± 1.5 N [1000], 7.1 ± 1.5 N [1100], and 15.3 ± 1.5 N [1110] (Fig. 3H). Based on these values, we selected a servo with a peak output force above 15 N, which is sufficient to meet the highest demand while remaining below the force needed to deform a fully jammed (locked) segment.

Despite operating at matched tail amplitudes, each gait produced distinct kinematics (Fig. 3I and movie S3). Midcycle midline profiles and the corresponding peak amplitude along the body differ systematically across gaits (Fig. 3, J and K). Joint bending angle θ_Joint_ measurements confirmed mode-specific bending patterns: In [0000] mode, the four joint angles were 10.1° ± 0.63°, 10.75° ± 0.32°, 9.9° ± 0.25°, and 10.36° ± 1.21° (from head to tail), whereas in [1110] mode, they were 2.2° ± 1.1°, 2.3° ± 1.1°, 3.4° ± 1.8°, and 37.6° ± 1.8°, respectively (fig. S14). These results confirm that our robotic fish can swiftly transition among anguilliform-like [0000], subcarangiform-like [1000], carangiform-like [1100], and thunniform-like [1110] gaits by programmatically tuning joint rigidity. This ability to lock or unlock segments effectively mimics biological muscle recruitment for adaptive robotic locomotion.

### Multimodal swimming of the robotic fish

**Fig. 4.**
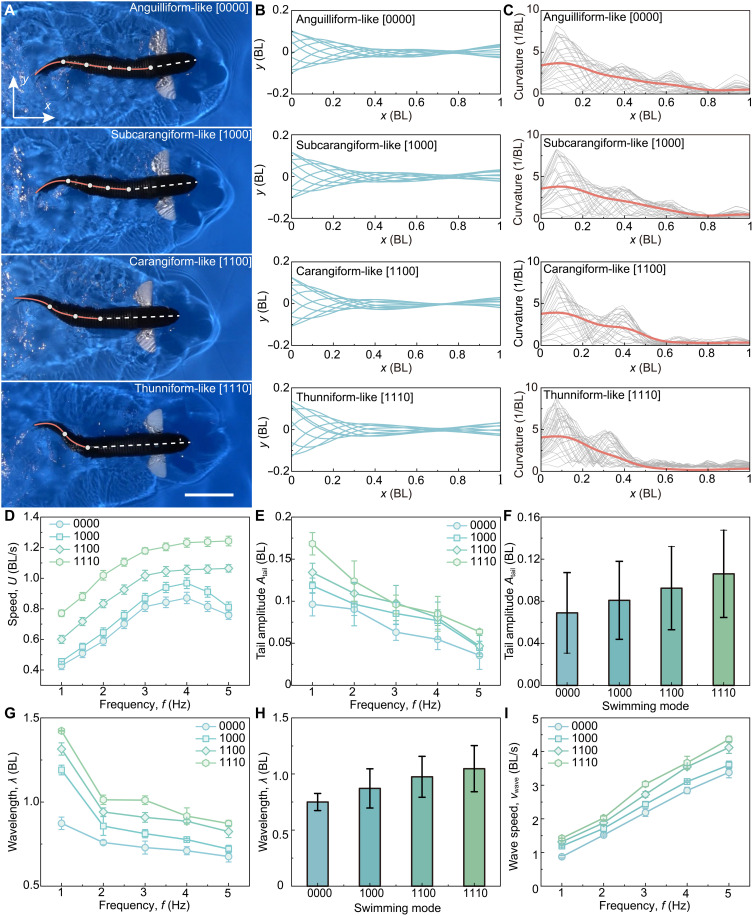
Swimming performance and kinematics of the multimodal robotic fish. (**A**) A snapshot of the robotic fish demonstrating four distinct swimming modes at a driving frequency *f* of 2 Hz (movie S4). Scale bar, 20 cm. (**B**) Body wave profiles of the robotic fish shown in (A), captured at 10 equally spaced time intervals during a single tail-beat cycle at 2 Hz. (**C**) Body midline curvature profiles along the fish length; red lines indicate the mean curvature. (**D**) Swimming speed *U* across the four modes at various frequencies *f* (movies S5 and S6) (*n* = 3). (**E**) Tail-tip amplitude *A*_tail_ versus *f* (*n* = 3). (**F**) The average *A*_tail_ under different modes (*n* = 5). (**G**) Changes in wavelength λ with frequency *f* for the four modes (*n* = 3). (**H**) The average wavelength λ under different modes (*n* = 5). (**I**) Frequency dependence of wave speed *v*_wave_ among four gaits (*n* = 3). All error bars represent ±1 SD.

By sequentially locking its tensegrity joints, the robotic fish demonstrates four distinct locomotion gaits, namely anguilliform-like [0000], subcarangiform-like [1000], carangiform-like [1100], and thunniform-like [1110] (Fig. 4A, fig. S15, and movie S4). The robot also switches between these modes rapidly. For example, the transition from the fully compliant [0000] gait to the most rigid [1110] gait takes under 1 s (fig. S16). Figure 4B shows the midline kinematics of these four gaits at 2 Hz, each characterized by a distinct curvature profile along the body. The normalized curvature profiles further highlight these differences (Fig. 4C). Across all modes, the average curvature increases toward the tail. Meanwhile, as the mode transitions from anguilliform-like [0000] to thunniform-like [1110], the anterior body’s curvature gradually decreases, reflecting reduced forebody undulation. In the anguilliform-like gait [0000], the entire body actively undulates, producing a relatively uniform curvature distribution along its length. In the other three gaits, the undulation is more localized, with the curvature peaks concentrated in the posterior body and caudal fin regions, which serve as the primary sites of flexion. Furthermore, the midline kinematics of our robotic fish closely matches that of real biological fish (fig. S17), confirming the successful replication of the four distinct swimming gaits observed in nature.

During free-swimming tests, all modes rapidly accelerate to a steady-state speed. We define the steady speed *U* as the average velocity over the final 40% of a stable swim (fig. S18). Under the same driving frequency *f* and prescribed tail amplitude *A*_tail_, the swimming speed *U* increases as the number of locked joints (Fig. 4D and movie S5). In other words, modes with stiffer front sections (closer to thunniform) achieved higher speeds. The relationship between *U* and *f* differs markedly among the gaits (movie S6). For the carangiform-like [1100] and thunniform-like [1110] modes, *U* increases with *f* over the tested range. The highest speed observed was about 1.24 BL/s (~0.81 m/s), achieved at 5 Hz in the thunniform-like mode [1110]. In contrast, the anguilliform-like [0000] and subcarangiform-like [1000] modes exhibit a nonmonotonic speed-frequency response. Their speeds initially increase with *f*, reaching peaks around 4 Hz (0.87 BL/s or 0.57 m/s for [0000] and 0.96 BL/s or 0.62 m/s for [1000]), but then decline as the frequency continues to rise beyond this point.

We analyzed the body kinematics in detail to understand why the anguilliform-like mode loses speed at high frequency, whereas the thunniform-like mode continues to improve. Midline profiles over one cycle from 1 to 5 Hz (fig. S19) show that, from [0000] to [1110], anterior undulation is progressively suppressed while lateral motion localizes to the peduncle and caudal fin, consistent with the curvature patterns in Fig. 4C. Increasing frequency *f* causes the body wave to contract spatially and the in-water amplitude to diminish, reflecting stronger fluid loading at higher *f*.

We then quantified the realized tail amplitude *A*_tail_. We found that hydrodynamic damping reduces the *A*_tail_ relative to the commanded value, with stronger attenuation at higher *f* (Fig. 4E). This attenuation is gait dependent: Modes with more locked joints preserve a larger fraction of the intended motion. The mean *A*_tail_ averaged over 1 to 5 Hz increases systematically from [0000] to [1110] (Fig. 4F). Joint locking also influenced the body-wave wavelength (λ). Across all gaits, λ shortened systematically as *f* increased (Fig. 4G), indicating a contracted wave at higher tail-beat rates. However, at a given frequency, the wavelength was longer in modes where undulation was concentrated near the tail. The mean λ (1 to 5 Hz average) was 0.75 ± 0.08 BL for [0000], 0.87 ± 0.17 BL for [1000], 0.97 ± 0.18 BL for [1100], and 1.05 ± 0.21 BL for [1110] (Fig. 4H). This trend shows that concentrating deformation near the tail produces a longer body-wave wavelength. Notably, it mirrors the pattern in biological counterparts. In nature, anguilliform swimmers have a shorter λ (around 0.75), whereas thunniform swimmers have a longer λ (around 1.14), which reinforces that our platform faithfully reproduces the four distinct swimming gaits found in nature (*65*). Because the wave propagation speed is given by *v*_wave_ = *f*λ, wave speed increases approximately linearly with *f* and it is higher for gaits from [0000] to [1110] (Fig. 4I and fig. S20).

At a given frequency *f*, the robot’s forward speed *U* is primarily determined by the realized tail amplitude *A*_tail_ and the wave speed *v*_wave_. Both of these factors increase as the gait becomes more thunniform-like (from [0000] to [1110]), explaining the speed ranking observed in Fig. 4D. As *f* increases from 1 to 5 Hz, *A*_tail_ tends to attenuate (due to added fluid damping) while *v*_wave_ rises. In the posterior-biased gaits ([1100] and [1110]), the boost in wave speed dominates, so *U* continues to grow with frequency. In the more compliant gaits ([0000] and [1000]), however, the shrinking *A*_tail_ leads to a nonmonotonic relationship between *U* and *f*. These observations motivated the hydrodynamic analysis that follows.

### Hydrodynamic analysis of multigait swimming

**Fig. 5.**
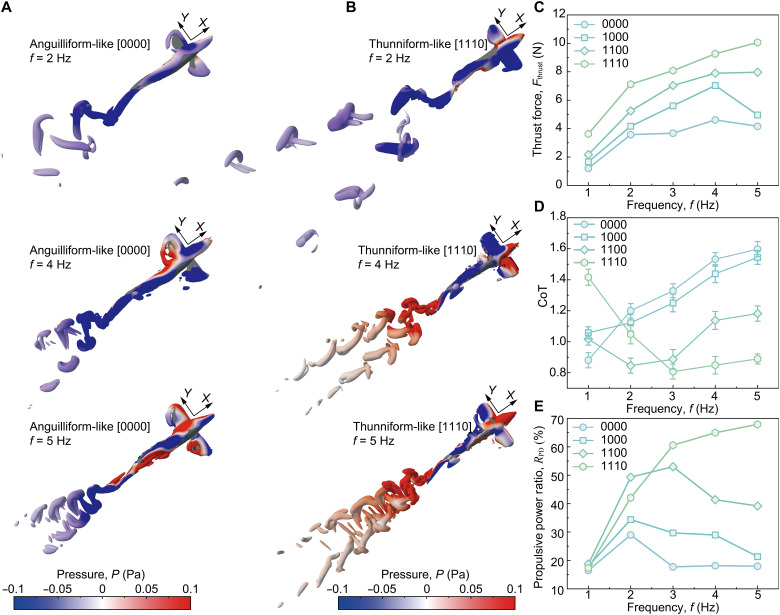
Hydrodynamic analysis of the multimodal robotic fish. (**A** and **B**) Pressure isosurfaces on a longitudinal plane, colored by pressure *P* (pascal) for two swimming gaits: (A) anguilliform-like [0000] and (B) thunniform-like [1110]. From top to bottom: *f* = 2, 4, 5 Hz, highlighting reduced lateral dipoles and a stronger axial jet in the more posterior-biased modes (*Q* = 15). (**C**) Thrust force *F*_thrust_ from CFD versus frequency *f*. (**D**) Cost of transport (CoT = *P*_in_/mg*U*) versus *f* (*n* = 3). (**E**) Propulsive dissipative power ratio (*R*_PD_ = *P*_T_/*P*_diss_) versus *f*. All error bars represent ±1 SD.

As illustrated in Fig. 5 (A and B) and figs. S21 and S22, the CFD simulations reveal distinct wake vortex structures and pressure distributions across the four swimming gaits at 2 Hz (movie S7). In the thunniform-like [1110] gait, large-amplitude tail flapping promotes strong vortex generation and shedding, which, in turn, imparts a substantial reaction force on the body. Concentrating undulation at the tail also creates a large pressure difference along the body surface, boosting thrust. As a result, the thunniform-like [1110] gait reaches the highest swimming speed, as also evidenced by the increased vortex spacing. By contrast, the anguilliform-like [0000] gait sheds wake vortices that quickly break up, causing momentum to disperse in multiple directions and part of the energy to be dissipated as lateral disturbances. This reduces forward thrust and efficiency. The enhanced asymmetry between the two vortex rows amplifies lateral forces and asymmetric perturbations, thereby facilitating maneuvering behaviors such as turning.

We further compare the pressure distributions of the four gaits at different flapping frequencies (Fig. 5, A and B, and fig. S22). As the frequency increases from 2 Hz, then 4 Hz to 5 Hz, four gaits exhibit intensified trailing-edge vortices and an enlarged pressure differential along the body, which generally enhances thrust production. However, at 5 Hz, the [0000] gait develops a high-pressure region near the head and pectoral fins. This adverse pressure concentration counteracts the thrust gained from the tail, resulting in the thrust force that is smaller than that at 4 Hz. In contrast, the [1110] gait exhibits primary pressure amplification near the tail, thereby enhancing thrust and yielding higher thrust at 5 Hz than at 4 Hz.

Based on these observations, we calculated the time-averaged thrust (*F*_thrust_) for each gait using CFD (Fig. 5C). The computed thrust matches experimental trends. In the carangiform-like [1100] and thunniform-like [1110] gaits, thrust increases monotonically with frequency, reaching about 8.0 and 10.1 N, respectively, at 5 Hz. By contrast, the thrust in the [0000] and [1000] gaits peaks at 4 Hz (4.61 and 7.03 N, respectively) and then declines at 5 Hz. At 5 Hz, the [1110] gait generates 142% more thrust than the [0000] gait. These findings mirror the measured trends in swimming speed versus frequency and reinforce our earlier conclusion that a larger tail amplitude and higher wave speed produce greater propulsive force.

Next, we measured the input power *P*_in_ and defined the cost of transport (CoT = *P*_in_/*mgU*), a nondimensional metric representing the input power normalized by body weight (*mg*) and swimming speed (*U*) (Fig. 5D) (*58*). Distinct frequency-dependent trends in CoT emerged among the gaits. In [0000] and [1000], CoT rises steadily with frequency, from 0.89 and 1.07 at 1 Hz to 1.6 and 1.6 at 5 Hz, respectively. In [1100] and [1110], CoT follows a U-shaped curve, reaching its minimum 0.84 at 2 Hz and 0.8 at 3 Hz, respectively. Consequently, at 5 Hz, the [1110] gait has the lowest CoT, ~45% lower than [0000].

Last, we computed the propulsive power (*P*_T_ = *F*_thrust_*U*) to quantify the balance between propulsive and dissipative power for each gait (Fig. 5E) (*66*). Here, propulsive-dissipative ratio *R*_PD_ = *P*_T_/*P*_diss_, with dissipative power *P*_diss_ = *P*_in_ − *P*_T_. The thunniform-like [1110] gait achieves the highest efficiency, with the *R*_PD_ reaching 67.91% at 5 Hz. By contrast, the anguilliform-like [0000] and subcarangiform-like [1000] gaits peak much lower (28.99 and 34.37% near 2 Hz) and then decline at higher frequencies. This indicates that the thunniform gait is best suited for high-speed swimming. At high flapping frequencies, concentrating motion in the tail minimizes wasted energy moving the forebody and channels more energy into propulsion.

### Turning performance of the robotic fish

**Fig. 6.**
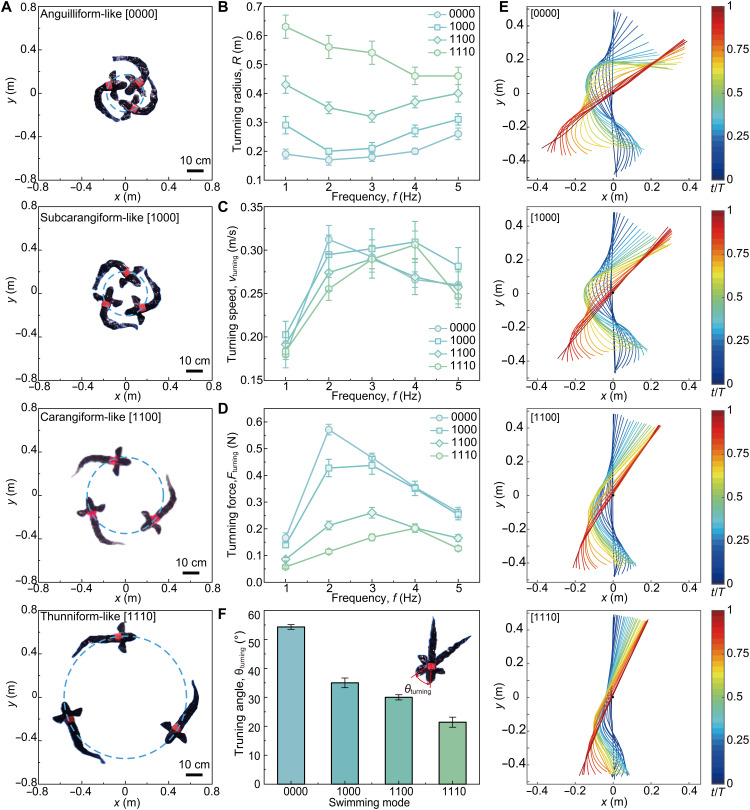
Turning performance of the multimodal robotic fish. (**A**) Snapshots of turning maneuvers for the four gaits; dashed circles indicate circles formed by the turn; scale bars, 10 cm. (**B**) Turning radius *R* across the four modes at various frequencies *f* (movie S8) (*n* = 3). (**C**) Turning speed *v*_turning_ versus *f* (*n* = 3). (**D**) Turning force *F*_turning_ versus *f* (*n* = 3). (**E**) Time-resolved midline shapes over one turning cycle for each gait; color indicates normalized time *t*/*T*. (**F**) Per-cycle turning angle θ_turning_ by gait, definition shown in the inset. All error bars represent ±1 SD.

Although the thunniform-like mode [1110] promotes efficient forward propulsion, relying solely on caudal fin oscillation could limit the robot’s flexibility in turning. To verify this hypothesis, we conducted turning experiments on the robotic fish. The robotic fish is able to use asymmetric tail fin strokes to generate lateral force and change its swimming direction. We found that the turning radius *R* depends on swimming modes and the tail-beat frequency (Fig. 6, A and B, and movie S8). At a given frequency, the turning radius shows the opposite trend to swimming speed, increasing as the number of locked joints increases (Fig. 6B). The smallest turning radius is 0.26 BL (0.17 m) at 2 Hz in the anguilliform-like mode [0000].

To understand why the anguilliform-like mode turns so much tighter, we examined potential factors. From the centripetal force relation, $R={\mathrm{mv}}_{\text{turning}}^{2}/F_{\text{turning}}$, where *m* is the robot’s mass, we can identify two key factors. One obvious factor is turning speed *v*_turning_, since, intuitively, a higher turning speed might require a larger radius. However, our experiments show that *v*_turning_ is not the dominant factor (Fig. 6C). For a given mode, *R* and *v*_turning_ often change in opposite directions as *f* varies (fig. S23). For instance, at 2 Hz, the [0000] mode has the highest *v*_turning_ (~0.31 m/s) yet the smallest turning radius, whereas the [1110] mode turns more slowly (~0.26 m/s) but with a much larger radius. This result indicates that simply reducing speed will not substantially improve the turning of a thunniform-like gait, and other factors play a larger role. We conclude that difference in *R* is governed more directly by the turning force *F*_turning_. As expected from its tight turns, the [0000] mode generates the highest lateral force, peaking around 0.57 N at 2 Hz (Fig. 6D). This substantial force enables rapid changes in direction. In contrast, the [1110] mode produces a much smaller peak *F*_turning_ (0.20 N at 4 Hz), which limits its ability to alter course effectively.

The reason for this disparity in force generation lies in the body kinematics during turning (Fig. 6E). The anguilliform-like [0000] mode involves pronounced bending along the entire body, resulting in a large body turning angle θ_turning_ of 54.3° (Fig. 6F). As more anterior joints are locked (progressing from [0000] toward [1110]), the body becomes progressively stiffer and the turning angle shrinks: 35° in the subcarangiform-like [1000] mode, 30° in the carangiform-like [1100] mode, and only 21.4° in the thunniform-like [1110] mode. The anguilliform-like mode recruits the entire body and produces larger lateral forces (turning force), generating tighter turns.

These results illustrate a clear performance trade-off. A higher number of locked joints (as in the thunniform-like mode [1110]) yields faster forward motion but at the cost of maneuverability (larger *R* and smaller θ_turning_). Conversely, fewer locked joints (anguilliform-like [0000]) sacrifice top speed for enhanced agility, allowing much tighter turns (smaller *R*). Thus, an effective strategy is to dynamically switch between swimming modes to meet different task requirements. Each mode emphasizes either maneuverability or speed (or a balance of both), similar to the spectrum of locomotion seen in real fish. For instance, the anguilliform-like mode [0000] uses eel-like undulations of the entire body and is well suited for scenarios requiring high agility and tight maneuvering (navigating around obstacles). In contrast, the thunniform-like mode [1110] relies mainly on oscillating a stiff tail (as tunas do) to achieve efficient, high-speed cruising. Between these extremes, the robotic fish can use intermediate gait patterns, such as modes [1000] and [1100], which offer a balance of speed and maneuverability. These midrange modes sacrifice a bit of agility for more thrust (or vice versa), allowing the robot to optimize performance for mixed conditions. This adaptive approach mirrors the versatility observed in the locomotion of real aquatic animals, enabling the robotic fish to balance speed and maneuverability as needed.

### Adaptive multimodal swimming through real-time mode switching

**Fig. 7.**
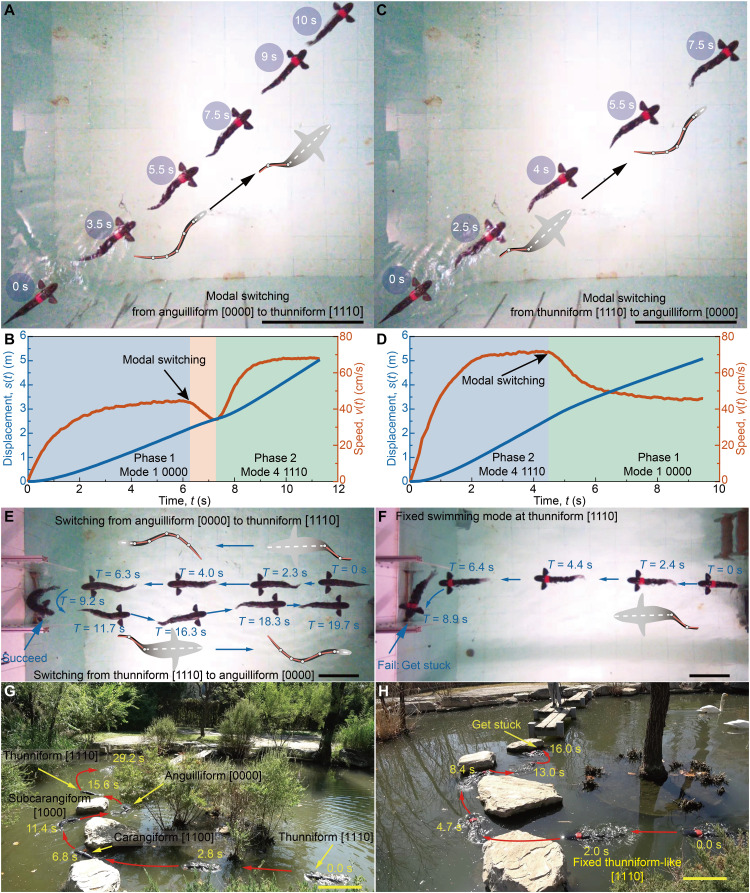
Multimodal swimming transition performance of the robotic fish. (**A** and **B**) Forward swimming with a mode transition from anguilliform-like mode [0000] to thunniform-like mode [1110] (A) and thunniform-like mode [1110] to anguilliform-like mode [0000] (B) (movie S9). Scale bar, 50 cm. (**C** and **D**) Evolution of the velocity *v*(*t*) and displacement *s*(*t*) as functions of time during the swimming process shown in (A) and (B), respectively. (**E**) Robotic fish achieving high-speed swimming and U-turn maneuvers in constrained environments via mode switching (movie S10). Scale bar, 50 cm. (**F**) Fixed thunniform-like mode fails to complete a U-turn in a constrained environment. Field trial demonstrating agile navigation through narrow rock crevices using mode switching in natural water (**G**) and single-mode operation results in entrapment in narrow crevices (**H**) (movie S11) Scale bar, 20 cm.

For a robotic fish to adapt to changing environments, the ability to switch locomotion modes in real time is essential. We demonstrate this capability by conducting experiments with our robotic fish in a laboratory water tank (movie S9). Initially, the fish swam in an anguilliform-like gait [0000] at a speed of 45 cm/s. At the 6.2-s mark, it executed a seamless transition to a thunniform-like mode [1110], completing this change in 1 s and doubling its speed to 70 cm/s by the 10th second (Fig. 7, A and B). The reverse transition—from thunniform-like back to anguilliform-like—at 4.2 s occurred even faster (within 0.5 s), resulting in a brief deceleration during the mode change (Fig. 7, C and D).

To highlight the advantages of mode switching, we tested the fish in a confined environment by introducing a 60-cm-wide U-shaped channel (narrower than the robot’s 65-cm length) into the tank (movie S10). In thunniform-like mode [1110], the fish approached the channel at high speed and switched to anguilliform-like mode [0000] at 4.0 s, allowing it to complete a 2-s U-turn before accelerating away in thunniform-like mode (Fig. 7E and movie S10). In contrast, a control trial with the fish locked in thunniform-like mode [1110] resulted in it becoming trapped at 8.9 s due to insufficient maneuverability (Fig. 7F).

Field trials further validate the system’s adaptability (movie S11). In the setting of Fig. 7G, the robotic fish rapidly approached natural rock formations in thunniform-like mode [1110], switched to carangiform-like mode [1100] at 2.8 s and subcarangiform-like mode [1000] at 6.8 s to negotiate a broad turn, and then shifted to anguilliform-like mode [0000] at 15.6 to navigate a narrow crevice, before reverting to thunniform for a rapid exit. By contrast, with the fish locked in thunniform-like mode [1110] (Fig. 7H), it could traverse only wider gaps and repeatedly collided with or became trapped in narrower passages. These experiments demonstrate the enhanced adaptability conferred by the robot’s multimodal swimming system in both controlled and natural aquatic environments.

## DISCUSSION

In this work, we present a multimodal soft robotic fish capable of four distinct swimming modes by emulating the full spectrum of fish-like gaits using tensegrity structures and BJLMs. By programmatically locking and releasing specific tensegrity joints, the robot seamlessly shifts between anguilliform (all segments soft, [0000]), subcarangiform (first joint locked, [1000]), carangiform (first two locked, [1100]), and thunniform (first three locked, [1110]) modes. This ability to actively tune body stiffness and activation length—analogous to fish muscle recruitment—enables the robot to mimic each gait’s kinematics precisely.

Traditional BCF robots are typically designed for a single swimming mode: Anguilliform robots use continuous flexible backbones (*46*–*49*), whereas thunniform robots feature rigid anterior bodies with specialized tail actuators (*56*–*59*, *67*, *68*). Such fixed architectures limit a robot to one gait and often require different hardware for different behaviors. Even designs described as “multimodal” remain limited compared with our posterior-biased gait transitions. Here, we compare our multimodal soft robotic fish with two previously reported multimodal swimmers: a dielectric elastomer actuator (DEA)-driven soft robotic fish (*60*) and the BCFbot robotic fish (*69*). The DEA platform demonstrated four modes with body-wave wavelengths of 1.20 BL, 2.74 BL, 2.58 BL, and 34.75 BL, respectively—values markedly different from those of real fish. Its fastest speeds were 0.28 BL/s in carangiform-like mode. Notably, the authors reported that the speed ranking is the reverse of the typical biological trend (speeds usually increase from anguilliform to thunniform). Furthermore, that robot required an external high-voltage power supply, meaning it swam tethered by cables. The BCFbot achieved three distinct BCF gaits. The corresponding body wavelengths were 0.16 BL, 0.30 BL, and 0.49 BL. In contrast, our robot’s performance spans the four BCF swimming gaits in one untethered platform. The forward speed increases from the anguilliform-like [0000] mode to thunniform-like [1110] mode. Likewise, the body-wave wavelength grows monotonically with joint locking: 0.75 ± 0.08 BL in [0000], 0.87 ± 0.17 BL in [1000], 0.97 ± 0.18 BL in [1100], and 1.05 ± 0.21 BL in [1110]. These values align closely with those of biological anguilliform, subcarangiform, carangiform, and thunniform swimmers, validating our robot’s bioinspired kinematics.

The BJLM is central to these mode transitions, providing rapid, large-range stiffness modulation. In its unjammed state, the BJLM is highly compliant, but vacuum activation quickly stiffens it (within 0.5 s), achieving a stiffness ratio of 46.60 ± 4.86. Compared to traditional stiffness-control methods, the BJLM offers superior responsiveness and a favorable decoupling of tensile and bending rigidity. For example, thermally activated low–melting point alloys require at least 150 s to adjust stiffness (*70*, *71*), and uniform fiber/layer jamming methods inherently couple bending stiffness (*72*–*74*). In contrast, the BJLM completes a full stiffness transition in under 0.5 s, and its bending rigidity increases only modestly (from 0.0135 to 0.1561 N/mm). By achieving a high tensile stiffness ratio while maintaining low bending stiffness, the BJLM overcomes a common trade-off, enabling the robotic fish to swiftly and accurately adjust its body-wave pattern across different swimming modes.

**Fig. 8.**
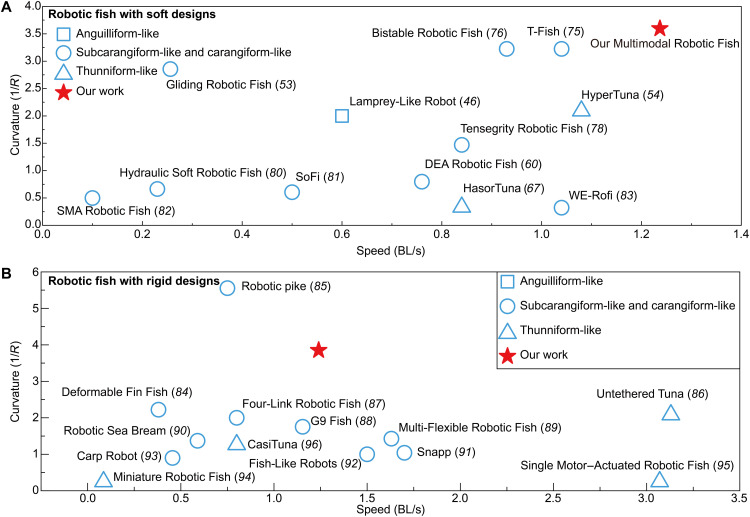
Performance comparisons with other robotic fish. Performance comparisons in terms of speed and turning radius of multimodal robotic fish with other (**A**) soft robotic fish and (**B**) rigid robotic fish. SMA, shape memory alloy; WE-Rofi, wire-driven elastic robotic fish.

These capabilities translate into substantial performance gains in speed and maneuverability. We compared our robot’s swimming speed and turning agility to those of previously reported soft robotic fish (Fig. 8A) (*46*, *53*, *54*, *67*, *75*–*83*). In thunniform-like mode, our fish reached a top speed of 1.24 BL/s, substantially faster than earlier multimodal designs. For instance, the fastest DEA-driven soft fish attained only 0.28 BL/s in carangiform mode and 0.17 BL/s in thunniform mode (*60*). Our maximum speed also surpasses single-mode robots: The Massachusetts Institute of Technology SoFi robot swims at 0.5 BL/s (*81*) and the HyperTuna at 1.08 BL/s (*54*). Maneuverability shows a similar advantage. Although DEA-driven multimodal fish did not report a turning radius, their earlier single-DEA soft fish achieved a turning radius of 1.5 BL (*79*), and the best previously reported result was 0.31 BL (*75*, *76*). In contrast, our robot achieves a tighter 0.26 BL turn in anguilliform-like mode, demonstrating superior agility.

We also compared our robot’s swimming speed and turning agility with a range of rigid-design robotic fish (Fig. 8B) (*84*–*96*). Rigid platforms tend to fall into two extremes: high-speed swimmers that can barely turn, and highly agile turners that are relatively slow. For example, one single-motor fish robot achieved 3.07 BL/s but required a 4-BL turning radius (*86*, *95*). Several sprint-oriented designs report no turning ability at all, including the Tuna Robotics platform (4.08 BL/s) (*56*), Tunabot Flex (4.6 BL/s) (*57*), an untethered bioinspired fish (3.8 BL/s) (*59*), iSplash-II (11.6 BL/s) (*97*), and a robotic flying fish (5.9 BL/s) (*98*). Conversely, agility-oriented systems can execute very tight turns but at low speeds. For instance, a robotic pike could turn within 0.18-BL radius at 0.75 BL/s (*84*, *85*), and a fish with deformable fins managed a 0.45-BL radius at 0.38 BL/s (*84*, *85*). Other designs occupy intermediate trade-offs. For example, four-link and three-link carangiform robots achieved turning radii of about 0.50 to 0.96 BL with speeds of 0.80 to 1.70 BL/s (*87*, *91*). Our multimodal soft robot breaks this speed-agility trade-off, occupying a new region of performance space. These comparisons clearly indicate that our multimodal design outperforms earlier soft robotic fish designs.

Beyond demonstrating engineering performance, our soft robotic fish serves as a robophysical model for studying undulatory BCF locomotion. We found that shifting the body’s undulatory deformation toward the tail enhances hydrodynamic thrust and efficiency: As the wave motion concentrates near the caudal fin, the wake contracts into tight, streamwise-aligned vortex pairs and pressure peaks develop at the tail. Accordingly, increasing the tailbeat frequency in posterior-biased gaits (carangiform-like and thunniform-like) yielded progressively higher thrust and lower CoT. At 5 Hz, for example, the thunniform-like mode produced 10.1 N of thrust (versus 8.0 N for the carangiform-like mode) and achieved the lowest CoT (0.89), attaining a peak propulsive efficiency of 68% (compared to 53% for the carangiform-like mode). These findings indicate that strongly posterior-biased kinematics maximize the conversion of body-wave energy into forward momentum while minimizing energetic losses, making such gaits optimal for high-speed cruising.

Turning performance exhibits the opposite trend. The anguilliform-like gait, which uses undulation of the entire body, achieved the tightest turns (0.26 BL at 2 Hz) by generating the highest lateral forces (0.57 N) and the largest turning angle (54.3° per tailbeat). In contrast, the thunniform-like mode could only turn with a much larger radius (0.55 BL) and produced a lower lateral force (0.20 N) along with a smaller turning angle (21.4°). Notably, these agility differences were driven not by tailbeat frequency (turning speed) but by the distribution of bending—greater body involvement in the undulation generated larger lateral forces.

This trade-off aligns with evolutionary adaptations among fish species. Anguilliform swimmers (eels) leverage full-body undulations for exceptional maneuverability in cluttered habitats, whereas thunniform swimmers (tuna) stiffen their anterior body to minimize head motion and maximize high-speed cruising efficiency. Our robotic fish mirrors these strategies to validate how fish balance speed and agility via muscle recruitment, bridging gaps in our understanding of biomechanical trade-offs.

The practical implications of seamless gait switching are substantial. The robot can autonomously toggle between a 1.24-BL/s cruising mode and a 0.26-BL turning mode, which suggests deployment versatility. For example, in reef monitoring or under-ice exploration, the anguilliform configuration’s flexibility would allow navigation through narrow crevices, while the thunniform configuration’s speed facilitates rapid transit between waypoints. By combining biomimetic reconfigurability with engineering robustness, this work paves the way for soft underwater robots capable of task-adaptive locomotion in varied environments.

While our multimodal soft robotic fish demonstrates exceptional versatility, speed, and maneuverability, several limitations remain. Current mode transitions rely on manual control rather than environmental feedback. Moreover, performance has only been validated in controlled settings, with real-world adaptability still to be explored. Future work will focus on enabling autonomous sensing-based control, hardware miniaturization, and robust field deployment.

## MATERIALS AND METHODS

### Fabrication of the BJLM

The SELs for the BJLM were fabricated using silicone rubber (Dragon Skin-20, Smooth-On), mixed at a 1:1 weight ratio. The degassed silicone was uniformly spread into a rectangular mold (mold 1: 10 mm by 10 mm by 0.1 mm) and leveled with a resin scraper. Polyester fibers were precisely embedded into the silicone using a custom fiber-winding machine (see fig. S3). Following the curing process, the composite laminate was cut into patches matching the required dimensions for specific SEL configurations. These patches were subsequently arranged within a second mold (mold 2: 10 mm by 10 mm by 0.5 mm), coated with EcoFlex 50 silicone, and then cured. They were subsequently trimmed into individual layers (10-mm wide, 0.5-mm thick), each incorporating discontinuous polyester fibers embedded in the silicone substrate. Multiple SELs were stacked and glued at both ends using Sil-Poxy silicone adhesive, with cornstarch applied to prevent unwanted adhesion between layers. Last, the assembled SEL stack was encapsulated in a silicone membrane and sealed securely with Sil-Poxy (for more manufacturing details, refer to text S3).

### Mechanical tests of the BJLM

Quasistatic tensile testing of the BJLM was performed using a Mark-10 F105 universal testing machine equipped with a 50-N load cell. Pull-to-failure and standard tensile tests were performed at an extension rate of 20 mm/min, while bending and buckling tests were conducted using a compression rate of 20 mm/min. Cyclic loading tests were conducted at an extension rate of 30 mm/min. Further experimental details and characterization results are provided in text S5.

### Swimming performance tests in water

Free-swimming trials were performed in a water tank (4 m by 5 m by 1.2 m). A global camera, mounted 3 m above the water surface, captured video footage at 30 frames per second. Due to differences in mechanical properties across swimming modes, distinct cable lengths were calibrated to maintain consistent tail kinematics. Specifically, cable lengths were set at 12.9 mm (anguilliform-like mode [0000]), 15.1 mm (subcarangiform-like mode [1000]), 18.6 mm (carangiform-like mode [1100]), and 25.4 mm (thunniform-like mode [1110]). Trials spanned a frequency range from 1 to 5 Hz. Postexperimental kinematic analysis and velocity measurements were conducted using Tracker software based on the recorded video frames.

### Extracting of body waves

To extract body wave data and locomotion characteristics, postprocessing of swimming videos involved sequential image processing and kinematic analysis. First, video sequences from free-swimming trials were decomposed into individual frames, generating a temporal image sequence of robotic fish locomotion. Each frame underwent grayscale conversion followed by adaptive binarization, where the background was rendered white, and the robotic fish appeared as a black silhouette through thresholding. The pixel coordinates of the robotic fish centroid were then extracted from the binarized images to track the planar position trajectories. Subsequently, the Canny edge detection algorithm was applied to identify left and right body contours from grayscale frames. Contour points were sampled at equidistant intervals along both lateral edges, and a cubic spline interpolation was used to reconstruct the midline—the neutral axis of body undulation. This neutral line represented the instantaneous body wave profile, serving as the basis for extracting kinematic parameters (e.g., wave amplitude).

We use a kinematic reconfiguration approach to decompose the whole-body motion of a robotic fish into periodic and secular components (*99*). First, raw body wave data in the x−y plane are rotated to align the mean velocity vector with the positive *u* axis (new axial direction), where the rotation angle θ is iteratively optimized via linear regression of periodic lateral $v_{\text{Periodic}}$ and residual axial $u_{\text{Res}}$ components until the angular change between iterations is $<10^{-2}\text{rad}$. The motion of midline body points in the *u*-*v* space is modeled as $v\left(t\right)=\xi_{0}^{v}+{\text{vel}}_{v}t+\frac{{\text{acc}}_{v}}{2}t^{2}+\sum_{i=1}^{2}A_{i}^{v}\text{cos}\left(i\omega t-\phi_{i}^{v}\right)$, combining nonperiodic terms (initial position, secular velocity/acceleration) and periodic harmonics (up to the second order). A basis matrix H=[1,t,sin(ωt),cos(ωt),sin(2ωt),cos(2ωt)] facilitates least-squares fitting to separate these components: Lateral motion includes both nonperiodic and periodic terms, while axial motion is simplified to a linear model $u\left(t\right)=\xi_{0}^{u}+{\text{vel}}_{u}t$ due to negligible periodic perturbations ($u_{\text{Periodic}}$ amplitudes <5% of body length). The iterative optimization loop compensates for residual axial-lateral coupling by updating θ using the slope from $v_{\text{Periodic}}=P_{1}u_{\text{Res}}+P_{2}$, ensuring physical consistency in motion decomposition. This framework enables the precise separation of kinematic components critical for analyzing robotic fish locomotion, with periodic lateral oscillations and secular axial drift robustly isolated through systematic parameter estimation and rotational alignment (see text S6 for details).

### CFD analysis

The 3D numerical model, with a total length *L*, is constructed based on the geometry of the experimental specimen. It includes a body trunk, a pair of pectoral fins, and a caudal fin, which is modeled as a zero-thickness membrane. The experimentally reconstructed deformations (fig. S19) under different modes and frequencies are prescribed as kinematic inputs to the model. The fish is allowed to adjust its streamwise (*x*-) velocity according to Newton’s second law, while other degrees of freedom are constrained.

A finite difference–based Cartesian grid immersed boundary method is used to solve the fluid-structure interaction problem, which involves large displacements and boundary deformations. The convective terms of the Navier-Stokes equations are discretized using a second-order Adams-Bashforth scheme, while an implicit Crank-Nicolson scheme is adopted for the diffusive terms. The model surface satisfies no-slip and no-penetration boundary conditions.

The overall computational domain is discretized using a nonuniform Cartesian grid with dimensions 15 *L* by 5 *L* by 5 *L*. A refined subdomain of 10.6 *L* by 1 *L* by 0.6 *L* is defined around the fish, with the finest grid resolution set to Δ*x* = Δ*y* = Δ*z* = 1/200 *L*. At the upstream boundary, a constant velocity condition is applied along with a homogeneous Neumann condition for pressure. At the downstream boundary, zero-gradient conditions are imposed for both velocity and pressure, while all remaining boundaries are subject to zero-stress conditions.

Time-dependent simulations are carried out until the fish reaches a steady cruising state. Grid convergence studies are performed with coarse, medium, and fine resolutions of Δ = 1/150 *L*, Δ = 1/200 *L*, and Δ = 1/250 *L*, respectively. Results from the medium and fine grids show close agreement. For instance, the mean swimming speed differs by less than 6% between the medium and fine resolutions.
