# Supplementary material for: Adaptive multimodal swimming gaits in a reconfigurable modular soft robotic fish
Source: Sci Adv. 2026 Jan 2;12(1):eaea1299. doi: 10.1126/sciadv.aea1299 (PMC12758535; doi:10.1126/sciadv.aea1299)
Supplement: Supplementary file 1 — Supplementary Text S1 to S6 Figs. S1 to S23 Legends for movies S1 to S11 [file sciadv.aea1299_sm.pdf]

Science Advances
AAAS

Supplementary Materials for

Adaptive multimodal swimming gaits in a reconfigurable modular soft robotic fish

Bo Wang *et al.*

Corresponding author: Junzhi Yu, yujunzhi@pku.edu.cn

Sci. Adv. **12**, eaea1299 (2026)
DOI: 10.1126/sciadv.aea1299

The PDF file includes:

Supplementary Text S1 to S6
Figs. S1 to S23
Legends for movies S1 to S11

Other Supplementary Material for this manuscript includes the following:

Movies S1 to S11

Text S1. Onboard vacuum-jamming stiffness control and antagonistic tendon actuation

Negative pressure for the bioinspired jamming layer muscles (BJLM) is provided by a miniature diaphragm vacuum pump (KLVP5-E-B24, KAMOR, China) integrated in the head (Fig. S1A and B). The pump connects to the sealed BJLM chambers through lightweight tubing and solenoid valves (H103, HIGHEND, China) as shown in Fig. S1B and D. Applying vacuum puts the BJLM in a jammed high-stiffness state. Venting to atmosphere returns it to a compliant state. By modulating the solenoid valve duty cycle and the pump on or off state, the vacuum level is controlled from 0 to −60 kPa gauge, enabling fast and reversible stiffness tuning.

Each tensegrity joint is actuated by a symmetric cable pair anchored on opposite sides. Reciprocating motor rotation winds one cable and unwinds the other, which bends the joint and shortens or lengthens the local BJLM when it is unjammed. Under vacuum jamming, shear friction between segmented layers increases markedly. Cable motion then produces negligible bending and the joint is effectively locked. A block diagram and wiring layout are provided in Fig. S1.

Text S2. Mold-cast flexible caudal fin with embedded carbon fiber reinforcement

The flexible caudal fin is produced utilizing a mold-casting process with silicone rubber (Dragon Skin Ecoflex 10), recognized as a biocompatible elastomer exhibiting a low Young's modulus (0.35 MPa) and remarkable elongation at break (>500%). This composition ensures outstanding flexibility and hydrodynamic compliance, making it suitable for undulatory propulsion. As depicted in Fig. S2A, the fabrication process initiates with the preparation of a two-part mold that delineates the fin's geometric profile, encompassing the trailing edge curvature and thickness gradient. The Liquid Ecoflex 10 base and curing agent are combined in a 1:1 ratio, degassed to remove air bubbles, and then poured into the mold. Before complete curing, a pre-cut carbon fiber plate (0.2 mm thickness, matching the fin's shape and dimensions) is integrated into the silicone matrix, strategically positioned along the longitudinal axis to furnish structural reinforcement without compromising edge flexibility (Fig. S2B). These carbon fiber inserts, characterized by a high stiffness-to-weight ratio, enhance the fin's longitudinal rigidity, preserving its geometric configuration during high-frequency oscillations and preventing excessive lateral twisting. The composite structure, which combines a compliant silicone matrix for adaptive deformation with a carbon fiber core for shape retention, mimics the natural "stiffness gradient" observed in biological fins, thereby facilitating controlled wave propagation and efficient force transmission during self-propelled swimming. After curing at room temperature, the fin is demolded, yielding a lightweight, durable propulsor with specifically tailored mechanical properties for robotic applications. The carbon fiber-reinforced region stabilizes the base for consistent actuation, while the peripheral silicone allows for flexible trailing-edge undulation to produce thrust (Fig. S2C).

Text S3. Fabrication of sels with discontinuous fiber embedding

The SELs for BJLM were fabricated by first mixing an elastomeric platinum-cure resin (Dragon Skin-20, Smooth-On) at a 1:1 weight ratio. The mixture was processed in a planetary mixer for 1 minute, followed by a 1-minute degassing period. The degassed silicone was subsequently evenly applied to a rectangular mold measuring 18 mm (length) × 12 mm (width) × 0.2 mm

(height), designated as mold 1, and smoothed with a resin scraper to ensure even dispersion (Fig. S3). To enable precise fiber embedding, a fiber-winding machine with tunable operational parameters was constructed (Fig. S4A). The fiber winder is composed of a linear slide with a ball screw and smooth shaft, equipped with a raft fishing reel for translating and distributing polyester continuous fibers, and a rotating winding mandrel. Each component is controlled by a connected 57-step motor, with one driving rotational motion and the other linear motion. By adjusting the motor speeds, the translation speed of the linear slide and the rotational speed of the spindle can be independently regulated. The Mold 1 (Fig. S4B) assembly was mounted onto the fiber-winding machine, and the spacing between fiber wraps can be controlled by maintaining a specific ratio between the rotational speeds of the two motors. For example, given a ball screw lead of 5 mm, setting the linear slide motor speed to 5 rpm and the spindle rotation speed to 50 rpm achieves a wrap spacing of 0.5 mm. We secured the polyester fiber (Fig. S4C) (Fujix King 90 Type, Japan) spool onto the raft fishing reel and connected it to the fiber-winding machine. Next, the machine wound polyester continuous fibers onto Mold 1 (Fig. S3). Then, the fully cured laminate composite material was cut off from the mold into smaller patches according to the required polyester fiber length for specific SEL configurations (Fig. S3). Arrange the patches on Mold 2 (10 mm (length) × 10 mm (width) × 0.5 mm (height)) based on the type of SEL, coat with silicone (EcoFlex 50, Smooth-On), let cure, and cut into layers width of 10 mm and a height about 0.5 mm. Each SEL had segments of polyester thread embedded discontinuously in a thin silicone substrate (Ecoflex 50, Smooth-On) (Fig. S3). Several SELs were stacked and glued together on both ends with a silicone-based adhesive (Sil-Poxy, Smooth-On). A small amount of cornstarch was applied to the surfaces of the SELs to prevent the adhesion of silicone. The bundle of SELs was put inside a silicone membrane and sealed with Sil-Poxy. Several designs were manufactured with different linear loading fractions of SEL (γ = 0, 60, 70, 80, and 100%) and various numbers of SELs per fiber (N = 3, 5, 7, and 9).

Text S4. Analytical model of jamming behavior

We propose a model of BJLM and perform parametric optimization to achieve a maximized stiffness variation ratio. In the unjammed state, the overall stiffness of the BJLM can be obtained by summing the stiffness contributions from individual layers and the sealed silicone envelope (Fig. S5):

$$k_u = Nk_f + k_m \tag{1}$$

where N denotes the number of SELs layers, k_f represents the stiffness of a single SELs layer, and k_m is the stiffness of the sealed silicone envelope (Fig. S5). The silicone envelope stiffness k_m is modeled as linear:

$$k_m = \frac{E_s A_m}{L} \tag{2}$$

where E_s is the Young's modulus of silicone, A_m is the cross-sectional area of the silicone envelope, and L is the layer length. The stiffness k_f is derived from a series configuration of k_s and k_p:

$$k_f = \frac{k_s k_p}{k_s + k_p} \tag{3}$$

k_s and k_p are also modeled as linear:

$$k_s = \frac{E_s A_c}{L(1-\gamma)} \tag{4}$$

$$k_p = \frac{E_p A_c}{L\gamma} \tag{5}$$

where E_p is the Young's modulus of polyester, A_c is the cross-sectional area of the layer, and γ denotes the percentage of polyester length relative to the total fiber length.

In the jammed state, the BJLM is modeled as a spring network, where the total stiffness corresponds to the equivalent stiffness of the entire network. The interfacial stiffness components k_{pp}, k_{ps}, and k_{pm} (representing polyester-polyester shear, polyester-silicone shear, and polyester-silicone envelope, respectively) are calculated as:

$$k_{pp} = \alpha \frac{GA_{pp}}{2t} \tag{6}$$

$$k_{ps} = \alpha \frac{GeA_{ps}}{t} \tag{7}$$

$$k_{pm} = \alpha \frac{GA_{pm}}{t} \tag{8}$$

where α is the effective contact ratio, G is the shear modulus of silicone, A_{pp}, A_{ps}, and A_{pm} are the shear areas of the respective interfaces, e accounts for incomplete shear activation between polyester and silicone springs, and t is the effective silicone thickness under shear:

$$t(\Delta P) = t_0 - t_0 \frac{\Delta P}{E_s} \tag{9}$$

where t_0 is the initial silicone thickness and ΔP is the negative pressure.

To calculate the stiffness of the BJLM, we employ an electrical resistance network analogy. Since the series-parallel rules for springs are inversely analogous to those for resistors, we define the reciprocal of spring stiffness as its "resistance" (Fig. S6). For illustration, a two-layer SELs structure is converted into a resistor network by replacing each spring stiffness k with a resistor of value $1/k$. Using Kirchhoff's Voltage Law (KVL)—the algebraic sum of potential differences around any closed loop equals zero—we formulate KVL equations for three independent loops:

$$(I_1 - I_2)\frac{1}{k_{ps}+k_s} + (I_1 - I_3)\frac{1}{k_{pm}} = U_0 \tag{10}$$

$$I_2 \frac{1}{k_{pm}} + (I_2 - I_3)\frac{1}{k_{pp}} + (I_2 - I_1)\frac{1}{k_{ps}+k_s} = 0 \tag{11}$$

$$(I_3 - I_1)\frac{1}{k_{pm}} + (I_3 - I_2)\frac{1}{k_{pp}} + I_3 \frac{1}{k_{ps}+k_s} = 0 \tag{12}$$

Let the matrix representation be:

$$A=\begin{bmatrix} \frac{1}{k_{ps}+k_s}+\frac{1}{k_{pm}} & -\frac{1}{k_{ps}+k_s} & -\frac{1}{k_{pm}} \\ -\frac{1}{k_{ps}+k_s} & \frac{1}{k_{pm}}+\frac{1}{k_{pp}}+\frac{1}{k_{ps}+k_s} & -\frac{1}{k_{pp}} \\ -\frac{1}{k_{pm}} & -\frac{1}{k_{pp}} & \frac{1}{k_{pm}}+\frac{1}{k_{pp}}+\frac{1}{k_{ps}+k_s} \end{bmatrix} \tag{13}$$

where $I=\begin{bmatrix} I_1 & I_2 & I_3 \end{bmatrix}^T$, $U=\begin{bmatrix} U_0 & 0 & 0 \end{bmatrix}^T$. This system can be expressed as:

$$A \cdot I = U \tag{14}$$

$$I = A^{-1} \cdot U \tag{15}$$

$$I_1 = (A^{-1})_{11} \cdot U_0 \tag{16}$$

Solving for the equivalent resistance, we obtain:

$$R=\frac{U_0}{I_1}=\frac{1}{(A^{-1})_{11}} \tag{17}$$

Finally, by taking the reciprocal of the equivalent resistance:

$$\frac{1}{R}=(A^{-1})_{11} \tag{18}$$

The analytical expression for the stiffness of the two-layer structure is obtained as:

$$k=\frac{2k_{pm}(k_s+k_{ps})^2+2k_{pm}\left(k_s+k_{ps}\right)k_{pp}+2k_{pm}^2\left(k_s+k_{ps}\right)+k_{pm}^2k_{pp}+(k_s+k_{ps})^2k_{pp}}{2k_{pm}\left(k_s+k_{ps}\right)+2k_{pm}k_{pp}+k_{pm}^2+(k_s+k_{ps})^2+2\left(k_s+k_{ps}\right)k_{pp}}+k_m \tag{19}$$

Using this method, we calculated the stiffness of the three-layer SELs material, yielding:

$$k=k_m+k_n$$

where

$$k_n=\frac{1}{\frac{2k_p+k_{pp}+k_{ps}+k_s}{2k_pk_{pm}+2k_pk_{pp}+k_{pm}k_{pp}+k_{pm}k_{ps}+k_{pp}k_{ps}+k_{pm}k_s+k_{pp}k_s}+\frac{1}{k_p+k_{ps}+2k_s}} \tag{20}$$

Assuming the polyester fibers are inextensible (i.e., $k_p \to \infty$), the expression simplifies to:

$$k=k_m+k_{pm}+k_{pp} \tag{21}$$

Text S5. Mechanical tests of BJLM

To understand the mechanics of a single SEL, we first conducted pull-to-failure tensile tests (5 mm/min using a Mark-10 F105 fitted with a 50 N load cell) on individual SEL with varying polyester length percentages γ to understand how γ influences stiffness and maximum elongation before failure. Ten types of SEL, containing differing volume fractions of polyester γ, were studied: 0%, 60% type A, 60% type B, 70% type A, 70% type B, 80% type A, 80% type B,

90% type A, 90% type B, and 100%. Note that 0% and 100% are selected as control specimens 0% is pure elastomer; 100% is pure polyester thread. As shown in Fig. S8A, a force versus displacement plot of the tests reveals that the 0% SELs were the least stiff (initial stiffness of 0.0097 N/mm). As we can see, increasing the volume fraction of polyester increased stiffness, peaking at the pure polyester thread (initial stiffness of 2.9823 N/mm). As γ increases, so does the stiffness, which is consistent with our understanding of the mechanics of an SEL from the rule of mixtures. Average elongation at failure was calculated from the force-displacement curves (Fig. S8B). There is a monotonically increasing trend in elongations at failure. Starting at around 10 mm for 100%, elongation rises to 28 mm for 90% of both types, to 40 mm for 80% of both types, to 60 mm for 70% of both types, to 85 mm for 60% of both types, and then to 135 mm for 0%. SELs of the same polyester volume fraction exhibited similar elongations at failure, regardless of whether they were type A or type B. These results imply that BJLM, which is constituted of different amounts of type A and type B SEL, should have relatively homogeneous stiffness properties along their length.

With an understanding of the mechanics of a single SEL, we turn to test the BJLM themselves. To explore the design space for BJLM, we conducted unidirectional tensile tests on combinations of layer parameters. A universal testing machine (Mark-10 F105) was used to perform the tensile testing for BJLM of four different numbers of SELs (N = 3, 5, 7, 9) and four ratios of polyester (60%, 70%, 80%, and 90%). Each BJLM used in the tensile tests was 50 mm long. Five samples were produced for each type, and the force-displacement data for both the unjammed and jammed states of these individual samples were collected using the experimental setup. Initially, no vacuum pressure was applied, and each sample was stretched up to 1 mm at a rate of 5 mm/s before it was allowed to return to its relaxed state. This step was repeated for 5 cycles. Following this, the vacuum is then turned on to create vacuum pressures of 0 kPa, -10 kPa, -20 kPa, -30 kPa, -40 kPa, -50 kPa, and -60 kPa, respectively. The same samples were then stretched for an additional 5 cycles to collect the jammed force-displacement data, as plotted in Fig. S8C. Notably, as the absolute pressure value, number of SELs, and ratio of polyester increased, the BJLM's maximum load increased. From this test, we also learned that the force-displacement curves of BJLM are characterized by two stages. The first is that the force increases linearly with the displacement, in which the tensile strength is smaller than the static friction force between layers. The second is the nonlinear region, where the shear force in the interface exceeds the maximum static friction that can be sustained by the silicone interface between polyester threads in adjacent layers, leading to the slip of the SELs relative to one another.

We performed pull-to-failure tests on several specimens of the selected type (70% N9) to study the maximum elongation and force of the BJLM at different pressures (Fig. S7). As the absolute value of pressure increased, the maximum load sustained by the fiber also increased. The BJLM failure modes are characterized by three stages. The first is the slip of the SELs relative to one another, which caused subtle drops in force over a longer duration. The second is incremental individual SEL failure inside the BJLM. Incremental failure is evident in the frequent “stair-step” drops in force toward the end of each curve. The third stage of BJLM failure occurs when most of the individual SELs have failed. At this point, the elastomer sleeve supports the majority of the load.

In this work, we mainly focus on the linear region, where the BJLM will act like a stiff spring and store mechanical energy. Before slip, the slope of the linear region of force-displacement curves can be defined as the tensile stiffness of BJLM ($k = \Delta F/\Delta d$). The mean jammed stiffness of each combination is reported in Fig. S9 to visualize the various parameter contributions to stiffness (in N/mm). As we can see, a larger stiffness is required for a higher number of layers, higher pressure, as well as a higher polyester content (resulting in higher tensile stiffness for the layers). For example, increasing from $N = 3$ to $N = 9$ at $P = 60$ kPa and $\gamma = 70\%$ elevated the jammed stiffness by 400%. With $N = 9$ at $P = 40$ kPa, increasing from 60% to 70% increased the stiffness by 36%. Additionally, Figure S10 presents the ratio of jammed to unjammed stiffnesses. We chose to proceed with $\gamma = 70\%$ and $N = 9$ for subsequent testing and demonstrations because it exhibited the highest stiffness differential (46.60 ± 4.86) between the jammed and unjammed states. Such high stiffness modulation is critical for switching the robotic fish's locomotion modes, as each tensegrity joint can smoothly alternate between a compliant (unjammed) and a rigid, locked (jammed) state, directly mimicking biological muscle recruitment strategies.

The BJLM preserves low stiffness to bending and buckling in directions orthogonal to actuation, which is critical for maintaining joint flexibility during undulatory motion (Fig. S11). Linear fits of the force-displacement data yielded bending stiffness of 0.0135 N/mm (unjammed) and 0.1561 N/mm (jammed, -60 kPa), and buckling stiffness of 0.1612 N/mm (unjammed) and 1.3283 N/mm (jammed, -60 kPa). These values are orders of magnitude lower than the jammed tensile stiffness (25.56 N/mm), ensuring that bending resistance is negligible and does not interfere with normal body deformation when joints are unjammed.

We also tested the BJLM under cyclic loading to assess durability and dynamic response. In the first test, a BJLM was repeatedly stretched in the jammed state (-60 kPa, 11.3 N load) to simulate long-term locking. After 1000 cycles, the tensile force retention exceeded 75% (dropping from 11.3 N to 8.5 N), demonstrating robust stability under sustained load (Fig. S12). The force loss was primarily attributed to minor interfacial sliding between the SELs. Remarkably, however, when the BJLM transitioned from the jammed to the unjammed state, its original performance was fully restored. In the second test, the BJLM cycled between jammed and unjammed states for 100 cycles to mimic real-time mode switching. The force-displacement curves showed negligible change and consistent stiffness at each target displacement, confirming reliable reconfigurability without performance drift.

Text S6. Extracting of body waves

We employ a kinematic reconfiguration approach to decompose the whole-body motion of a robotic fish into periodic and secular components (*99*). Periodic components were extracted using a motion decomposition model, which distinguishes between non-periodic drift and periodic oscillations. For axial direction (u), dominated by non-periodic motion (linear velocity):

$$u(t) = \xi_0^u + \mathrm{vel}_u t + u_{\mathrm{Periodic}}(t) \tag{22}$$

where u_{Periodic} denotes residual periodic perturbation, typically negligible.

For the lateral direction v, motion is characterized by a coupling of non-periodic and periodic components. The lateral motion equation combines a quadratic non-periodic trend with harmonic periodic oscillations:

$$v(t) = \xi_0^v + \mathrm{vel}_v t + \frac{\mathrm{acc}_v}{2} t^2 + \sum_{i=1}^{2} A_i^v \cos\left(i\omega t - \phi_i^v\right) \tag{23}$$

The non-periodic components—comprising the initial lateral position ξ_0^v, uniform lateral translation $\mathrm{vel}_v t$, and gradual velocity change $(\mathrm{acc}_v/2)t^2$—describe smooth, non-repeating movements such as initial offsets, constant-speed drift, or acceleration/deceleration during turns. The periodic component incorporates two harmonics ($i = 1,2$): the fundamental frequency ($i = 1$) and its first harmonic ($i = 2$), both defined by the angular frequency $\omega = 2\pi f$ (where *f* is the tailbeat frequency), which captures rhythmic motions like tail or fin beats. Each harmonic is characterized by an amplitude (A_i^v), determining oscillation strength, and a phase (ϕ_i^v), dictating timing relative to a reference cycle. This formulation explicitly couples long-term drift with cyclic oscillations, enabling the separation of rigid-body translation (non-periodic) from deformational vibrations (periodic)—a critical distinction for analyzing biological locomotion (e.g., fish swimming) or robotic motion, where steady displacements and rhythmic appendage movements coexist to generate thrust and maneuvering forces.

For time-series data of each body point (*T* frames), a 6-column basis matrix H (Eq. 24) is constructed:

$$H = \begin{bmatrix} 1 & t_1 & \sin(\omega t_1) & \cos(\omega t_1) & \sin(2\omega t_1) & \cos(2\omega t_1) \\ \vdots & \vdots & \vdots & \vdots & \vdots & \vdots \\ 1 & t_T & \sin(\omega t_T) & \cos(\omega t_T) & \sin(2\omega t_T) & \cos(2\omega t_T) \end{bmatrix} \tag{24}$$

where rows correspond to time points t_1 to t_T, and columns encode: column 1 (non-periodic constant ξ_0^v), column 2 (linear velocity term *t*), and columns 3–6 ($\sin\omega t$, $\cos\omega t$, $\sin 2\omega t$, $\cos 2\omega t$ for $i = 1,2$ harmonics). Using least squares $C_v = \left(H^T H\right)^{-1} H^T v$, this matrix unifies 7 fitting parameters for lateral motion $v(t)$—$C_v = \left[\xi_0^v, \mathrm{vel}_v, \mathrm{acc}_v, A_1^v \cos\phi_1^v, A_1^v \sin\phi_1^v, A_2^v \cos\phi_2^v, A_2^v \sin\phi_2^v\right]^T$ —with the first 3 columns capturing non-periodic trends (initial position, linear velocity, quadratic acceleration) and the last 4 encoding periodic amplitudes/phases via sine/cosine decomposition.

In contrast, axial motion simplifies to 2 non-periodic terms ($u(t) = \xi_0^u + \mathrm{vel}_u t$, parameters $C_u = \left[\xi_0^u, \mathrm{vel}_u\right]^T$) due to negligible periodic residuals (<5% body length). For lateral periodic component $v_{\mathrm{Periodic}}(t)$, non-periodic terms (Columns 2–3 in H) are removed, retaining Column 1 (constant) and periodic Columns 3–6, yielding:

$$v_{\mathrm{Periodic}}(t) = \xi_0^v + \sum_{i=1}^{2} A_i^v \cos\left(i\omega t - \phi_i^v\right) = H\left(:,[1,3:6]\right) \cdot C_v\left([1,3:6]\right) \tag{25}$$

The axial periodic residual $u_{\text{Periodic}}(t) = u(t) - \left(\xi_0^u + \text{vel}_u t\right)$ serves exclusively for iterative rotation optimization, compensating minor axial-lateral coupling (via amplitude-limited residuals) to align the axial direction with mean velocity (free of periodic noise).
A four - step iterative loop with a convergence threshold of $\;=10^{-2}$ radians was employed to ensure that the axial motion u was aligned with the mean velocity, excluding periodic oscillations.
In each iteration k, the first step is the rotation operation. Based on the rotation angle $\theta^{(k-1)}$ obtained from the previous iteration, the original axial motion x and lateral motion y are rotated to obtain the new axial motion $u^{(k)}$ and lateral motion $v^{(k)}$. The calculation formulas are:

$$\begin{cases} u^{(k)} = xcos\theta^{(k-1)} + ysin\theta^{(k-1)} \\ v^{(k)} = xsin\theta^{(k-1)} + ycos\theta^{(k-1)} \end{cases} \tag{26}$$

The second step is the separation of motion components. For the axial motion, the axial velocity $\text{vel}_u^{(k)}$ in this iteration is calculated by taking the difference between the starting and ending values of $u^{(k)}$ over the entire time - series (with T frames) and dividing it by the difference in the number of frames, i.e., $\text{vel}_u^{(k)} = \left(u^{(k)}(T) - u^{(k)}(1)\right)/(T-1)$. For the lateral motion, it is decomposed into non - periodic and periodic parts:

$$v^{(k)}(t) = \xi_0^{v(k)} + \text{vel}_v^{(k)} t + \frac{\text{acc}_v^{(k)}}{2} t^2 + v_{\text{Periodic}}^{(k)}(t) \tag{27}$$

The third step is coupling compensation. By performing a linear regression of the lateral periodic component $v_{\text{Periodic}}^{(k)}$ against the axial residual $u_{\text{Res}}^{(k)} = u^{(k)} - \left(\xi_0^{u(k)} + \text{vel}_u^{(k)} t\right)$, the regression equation $v_{\text{Periodic}}^{(k)} = P_1^{(k)} \cdot u_{\text{Res}}^{(k)} + P_2^{(k)}$ is obtained. Then, the rotation angle is updated according to the regression coefficient $P_1^{(k)}$ as $\theta^{(k)} = \theta^{(k-1)} + \arctan\left(P_1^{(k)}\right)$ to compensate for the residual coupling between the axial and lateral motions.
The fourth step is the convergence check. If the absolute value of the difference between the rotation angle $\theta^{(k)}$ obtained in the current iteration and the rotation angle $\theta^{(k-1)}$ from the previous iteration is less than the threshold $\left|\theta^{(k)} - \theta^{(k-1)}\right| <$, the iteration is considered to have converged, and the iterative process is terminated.
Through this iterative process, the separation of the axial and lateral motions can be gradually adjusted, enabling the axial motion to more accurately reflect the mean velocity excluding periodic oscillations and improving the accuracy of motion analysis.

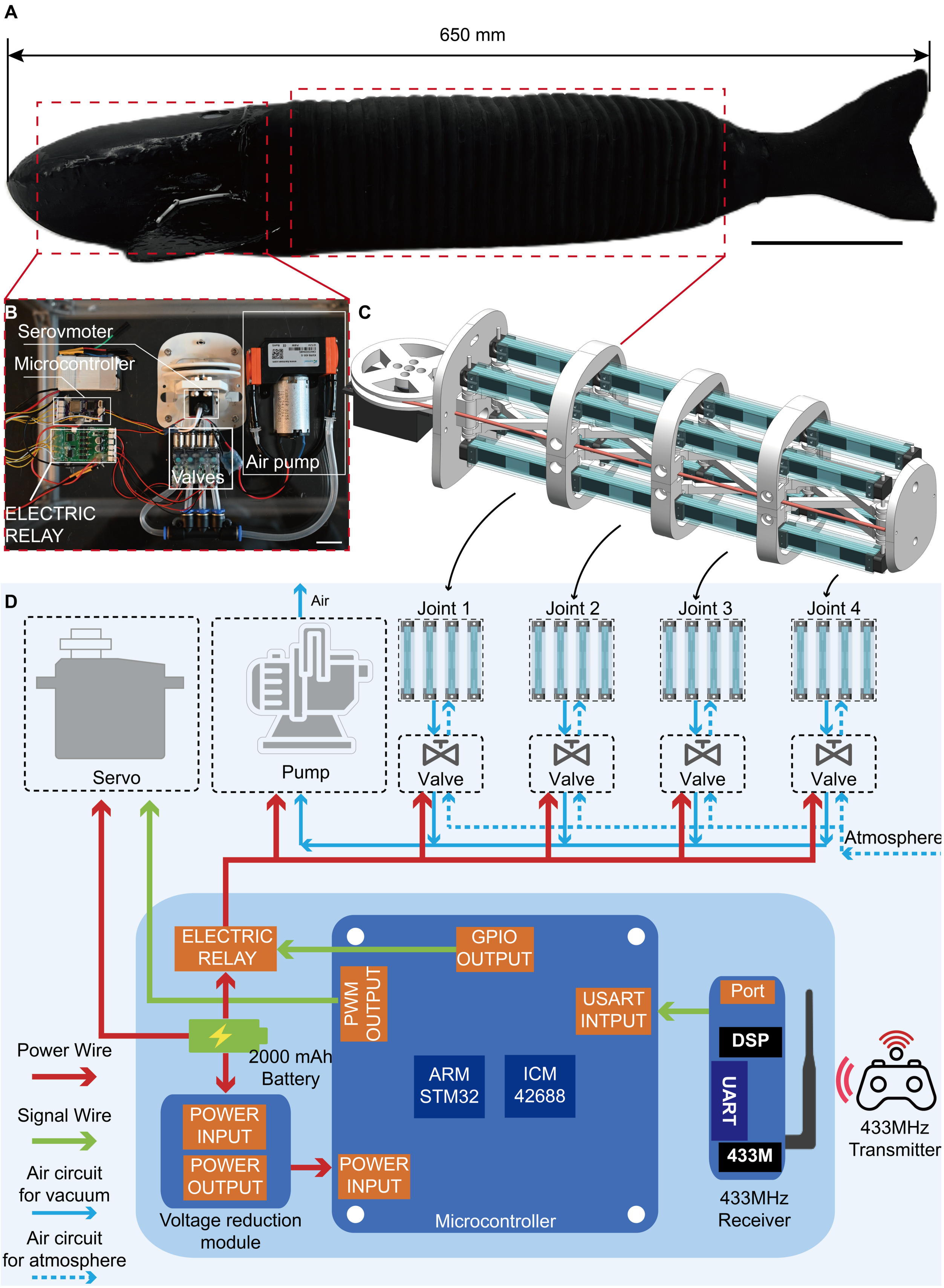

A
650 mm
B
Serovmoter
Microcontroller
Air pump
Valves
ELECTRIC
RELAY
C
D
Air
Joint 1
Joint 2
Joint 3
Joint 4
Servo
Pump
Valve
Valve
Valve
Valve
Atmosphere
ELECTRIC
RELAY
GPIO
OUTPUT
PWM
OUTPUT
USART
INTPUT
Port
DSP
UART
433M
2000 mAh
Battery
ARM
STM32
ICM
42688
POWER
INPUT
POWER
OUTPUT
POWER
INPUT
Voltage reduction
module
Microcontroller
433MHz
Receiver
433MHz
Transmitter
Power Wire
Signal Wire
Air circuit
for vacuum
Air circuit
for atmosphere

Fig. S1. Control and actuation system for the robotic fish. (A) Prototype of the robotic fish **(B)** Physical picture of electronic components. Scale bar, 20 mm. **(C)** Schematic diagram of the robotic fish tail section for undulation. **(D)**System diagram of the onboard electronics for control and actuation, as well as the robot's power supply. A cable-driven mechanism is adopted to bend the tensegrity body for swimming. Four valves are used to control the tensile stiffness of BJLM in each joint, enabling joint locking/unlocking. A negative pressure pump is connected to four solenoid valves, which in turn link to the BJLM units at the four joints.

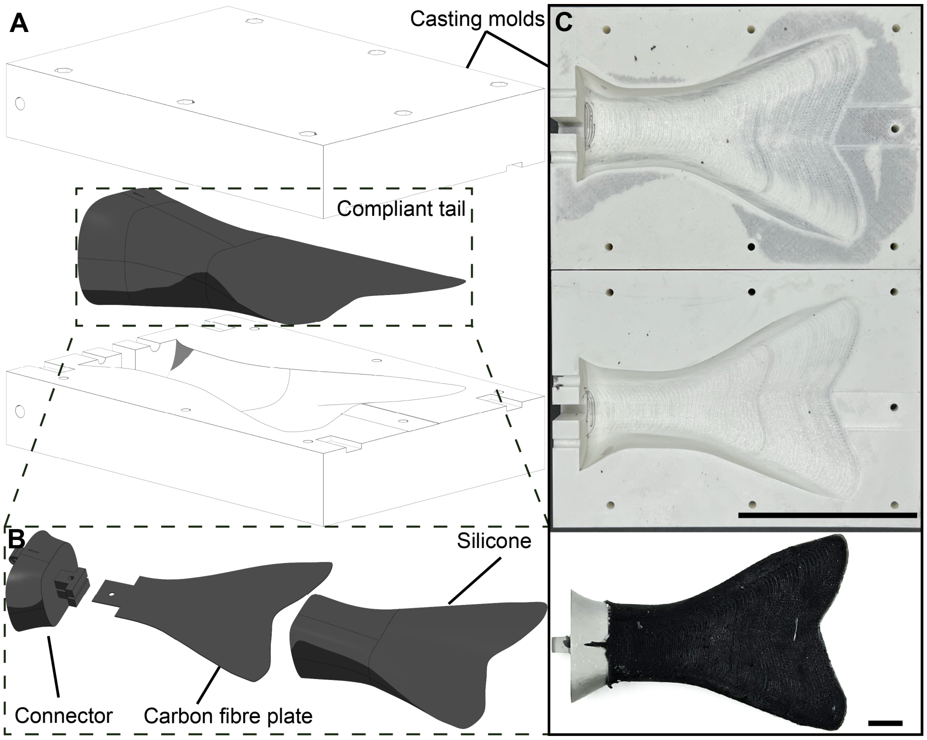

Fig. S2. Fabrication of the flexible caudal fin. (A) Mold-casting process of the flexible caudal fin using Ecoflex 10 silicone rubber. **(B)** 3D-printed mold for precise shaping of the fin geometry, including trailing edge curvature and thickness distribution. Scale bar, 10 cm. **(C)** Completed flexible caudal fin with embedded 0.2-mm-thick carbon fiber plate for structural reinforcement and configuration maintenance. Scale bar, 20 mm.

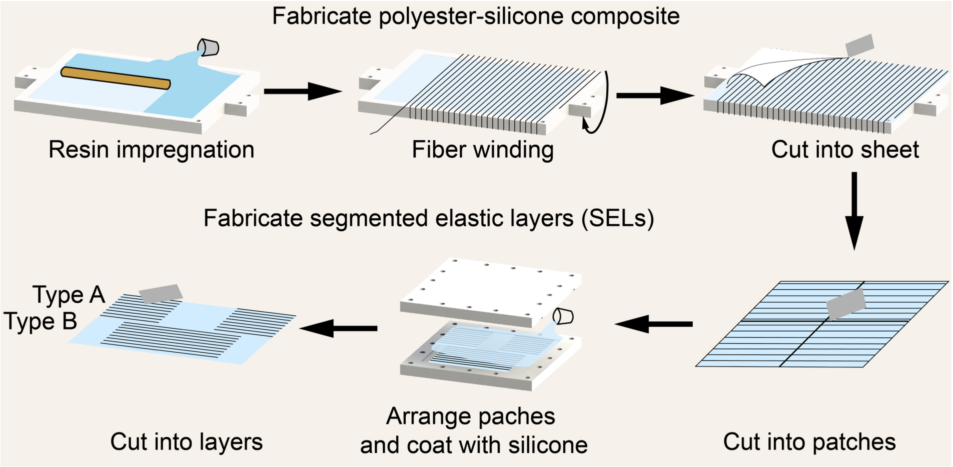

Fig. S3. Fabrication process of SELs. The mixture of 1A:1B (mass/mass) of the silicone (Dragon skin-20, Smooth-On) is put into a vacuum chamber with negative pressure to remove the bubbles and apply it evenly to the mold-1 (the grooves have dimensions of 10 mm × 10 mm × 0.1 mm). Next, mold-1 with silicone is mounted on a custom-made BJLM winding machine, and polyester threads are wound onto the rotating mold at 0.5 mm spacing. Then, the fully cured laminate material is cut off from the mold into smaller patches, with the size depending on the required polyester thread length for SEL. Arrange the patches on another mold (namely, mold-2), according to the type of SEL, coat with silicone (EcoFlex 50, Smooth-On), let cure, and cut into layers of width 10 mm (and height about 0.5 mm). Stack alternating type A and type B SELs, align them at both ends, and bond them with Sil-poxy. The length of polyester content is higher than 50% of the total SEL length within each type of SEL. This way, polyester sections will overlap when the two types of SEL are placed adjacent to each other in the silicone envelope.

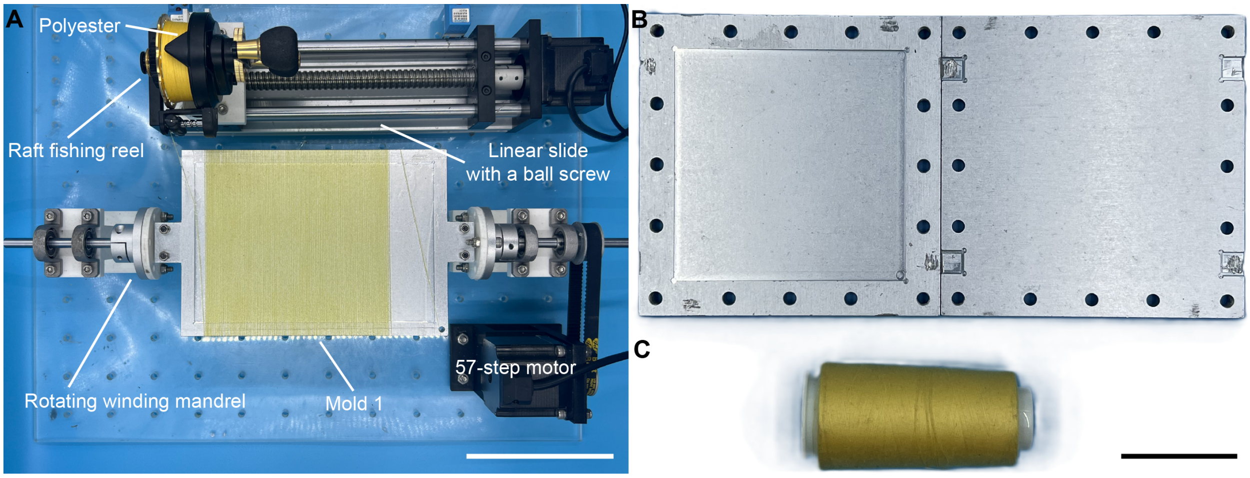

Fig. S4. Tools and molds for manufacturing SEL. (A) A custom fiber-winding machine enabled precise embedding of polyester fibers. Scale bar, 10 cm. **(B)** Rectangular mold (Mold 2) with dimensions: 10 mm (length) × 10 mm (width) × 0.5 mm (height). **(C)** Polyester fiber used: Fujix King 90 Type (Japan). Scale bar, 5 cm.

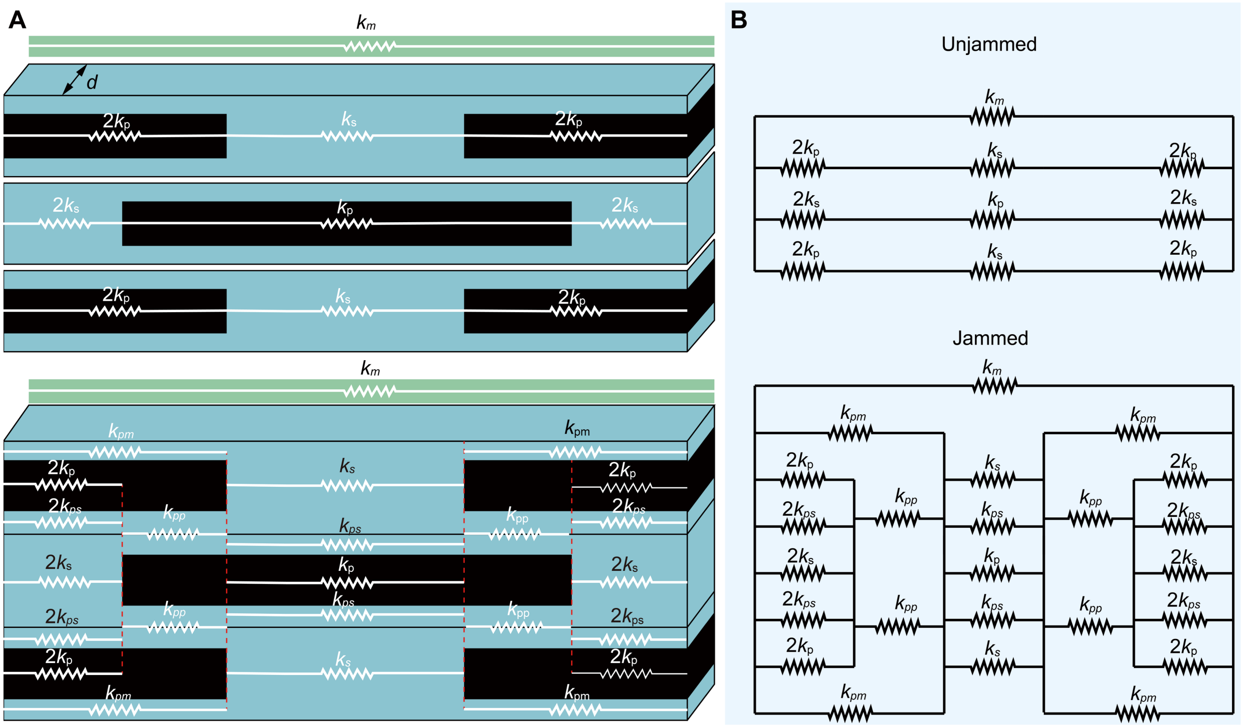

Fig. S5. Schematic of the analytical model of jamming. (A) schematic showing the BJLM behavior in the unjammed and jammed states. For clarity, only three SELs are demonstrated inside the BJLM. The equivalent spring constants of different sections are shown. **(B)** Spring model of the unjammed and jammed cases of a three-layer system.

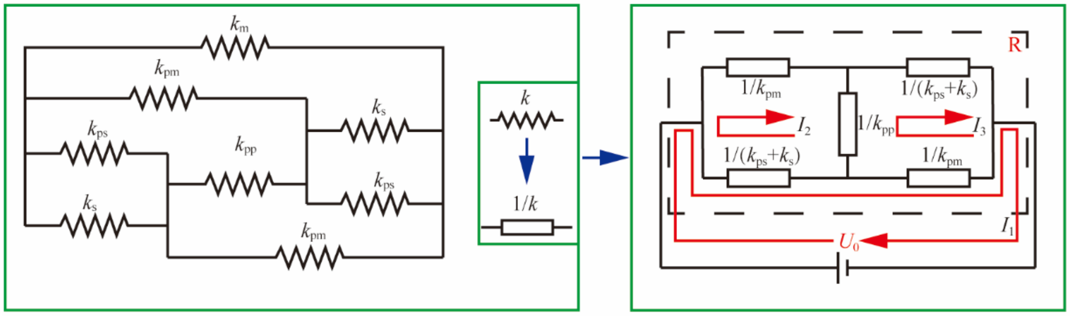

Fig. S6. Equivalent stiffness of the entire network. Simplified equivalent model of the jammed BJLM. The left side shows a two-layer model. Only the simplified jammed model for two layers of SELs is presented. Due to the bilateral symmetry, only half of the structure is presented.

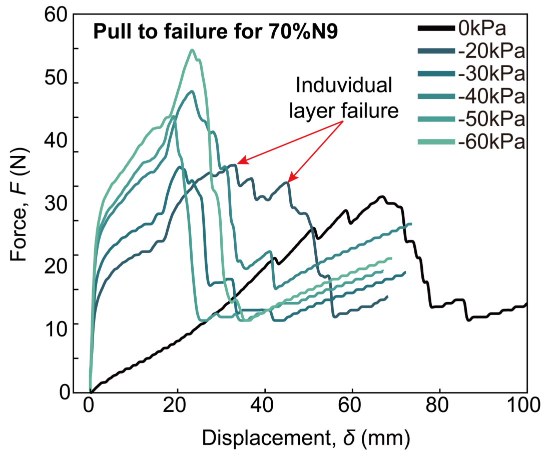

Fig. S7. Pull-to-failure test on 70% *N*9 BJLM at various pressures.

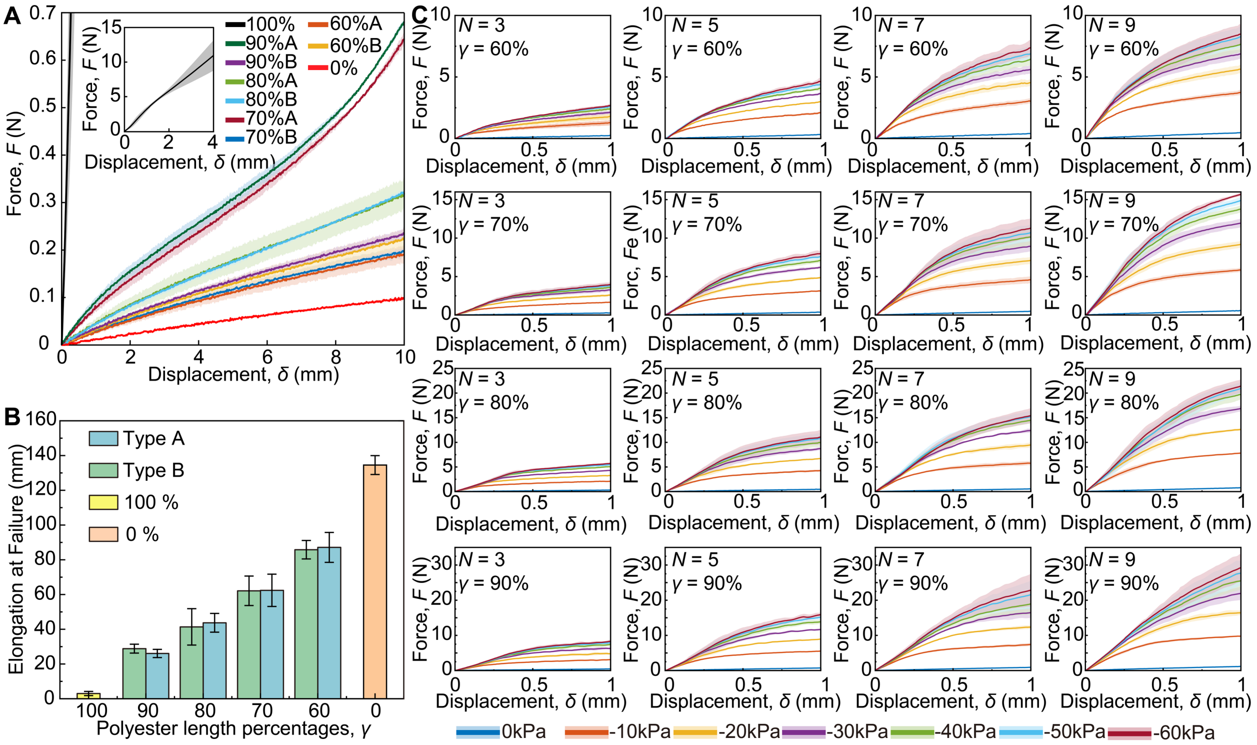

Fig. S8. Mechanical tests of SELs and BJLM. (A) Mean curves with standard deviation clouds for uniaxial tension tests of individual layers. The inset illustrates the small displacement regime to provide a clearer visualization of the mechanical behavior of polyester layers. Solid lines: mean response (n = 5); shaded areas: ± 1 SD. **(B)** Elongation at the failure of the various SEL specimens (n = 5). Error bars represent ±1 SD. **(C)** The force-displacement of BJLM at different numbers of SELs (N =3, 5, 7, 9) and vacuum pressures (0 kPa, -10 kPa, -20 kPa, -30 kPa, -40 kPa, -50 kPa, and -60 kPa), holding γ = 60%, γ = 70%, γ = 80%, and γ = 90%. Solid lines: mean response (n = 5); shaded areas: ± 1 SD.

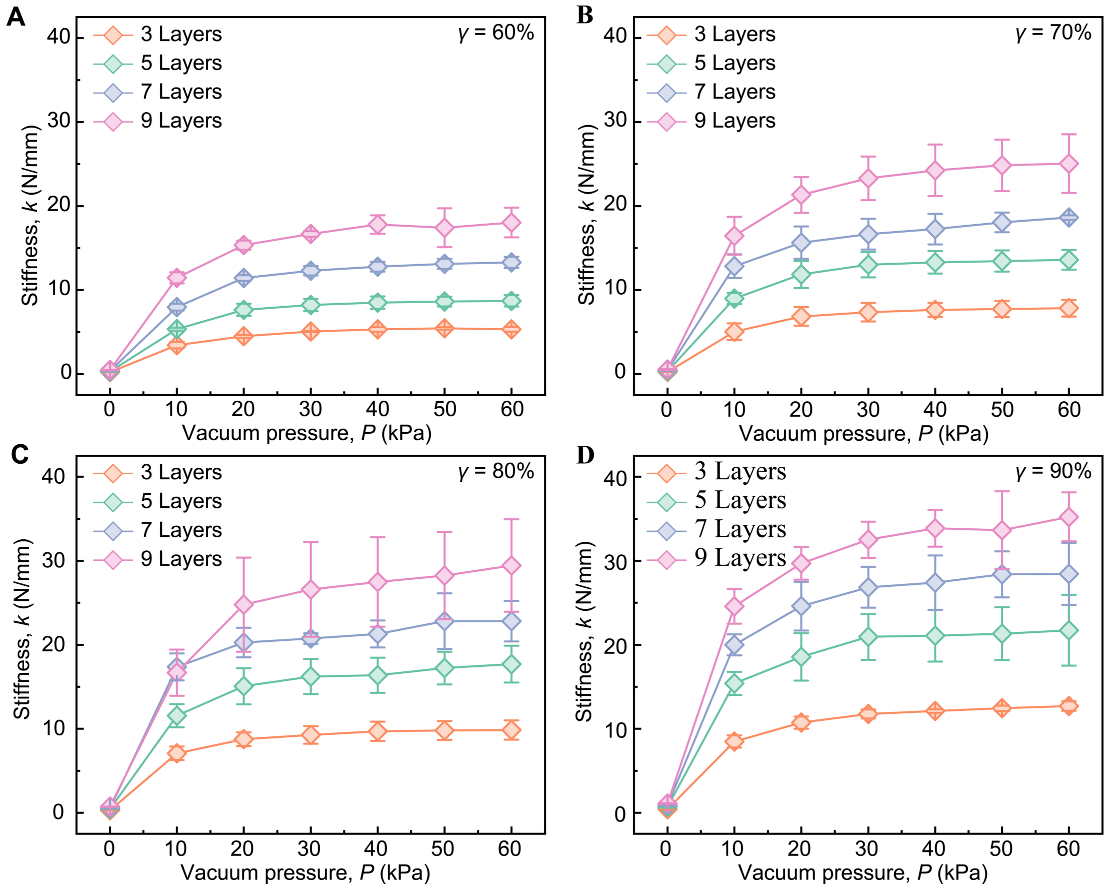

Fig. S9. The mean jammed stiffness of BJLM. The mean jammed stiffness k of BJLM at different numbers of SELs (N =3, 5, 7, 9) and vacuum pressures (0 kPa, -10 kPa, -20 kPa, -30 kPa, -40 kPa, -50 kPa, and -60 kPa), holding γ = 60% **(A)**, γ = 70% **(B)**, γ = 80% **(C)**, and γ = 90% **(D)**. The reported values represent the mean values of 5 samples at each design point. All error bars represent ±1 SD.

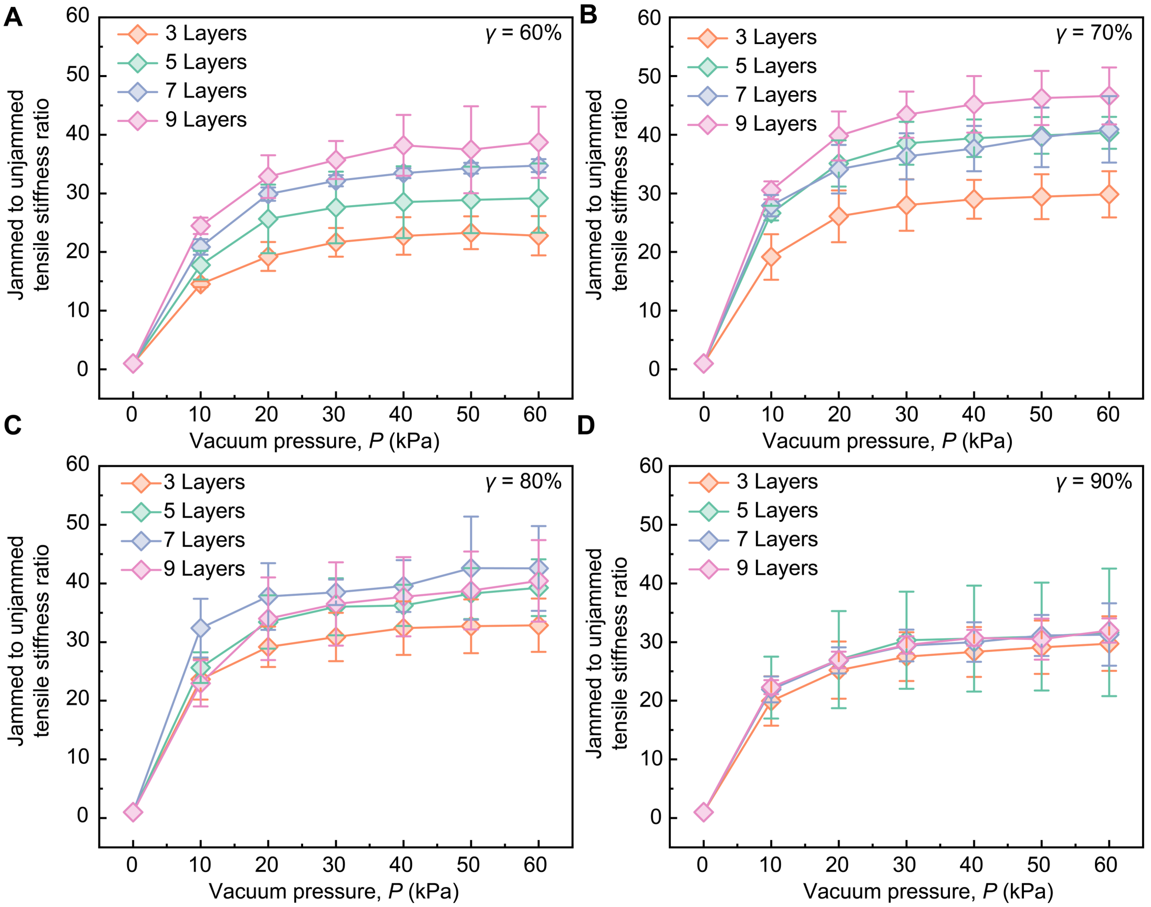

Fig. S10. The ratio of jammed to unjammed stiffness values of BJLM at different numbers of SELs (N =3, 5, 7, 9) and vacuum pressures (0 kPa, -10 kPa, -20 kPa, -30 kPa, -40 kPa, -50 kPa, and -60 kPa), holding γ = 60% (A), γ = 70% (B), γ = 80% (C), and γ = 90% (D). The reported values represent the mean values of 5 samples at each design point. All error bars represent ±1 SD.

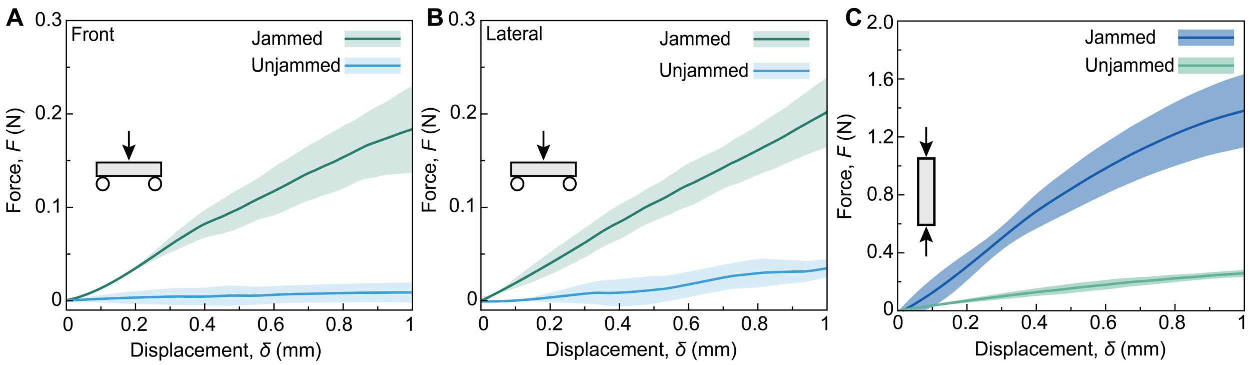

Fig. S11. Bending and buckling tests of the BJLM in jammed and unjammed states. (A) Bending tests of the BJLM in jammed and unjammed states. Solid lines: mean response (n = 5); shaded areas: ± 1 SD. **(B)** is test conducted with specimen rotated 90° from (A) Solid lines: mean response (n = 5); shaded areas: ± 1 SD. **(C)** Through-thickness compression showing the buckling response. Curves compare jammed (−60 kPa) and unjammed states. Solid lines: mean response (n = 5); shaded areas: ± 1 SD.

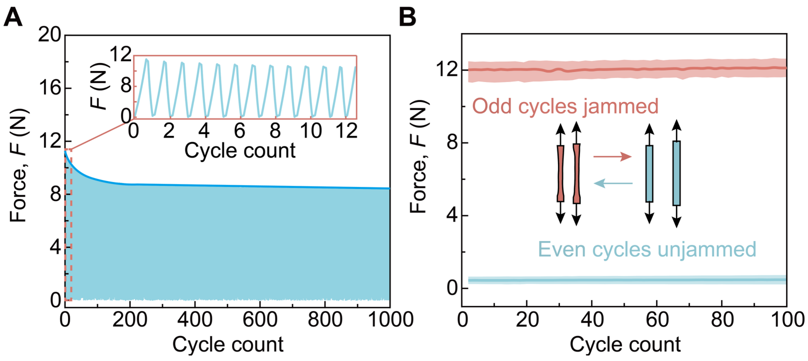

Fig. S12. Reliability tests: under 1000 tensile cycles in the jammed state (A) and under 100 cycles of alternating jammed/unjammed states (B). Solid lines: mean response ($n = 5$); shaded areas: ± 1 SD.

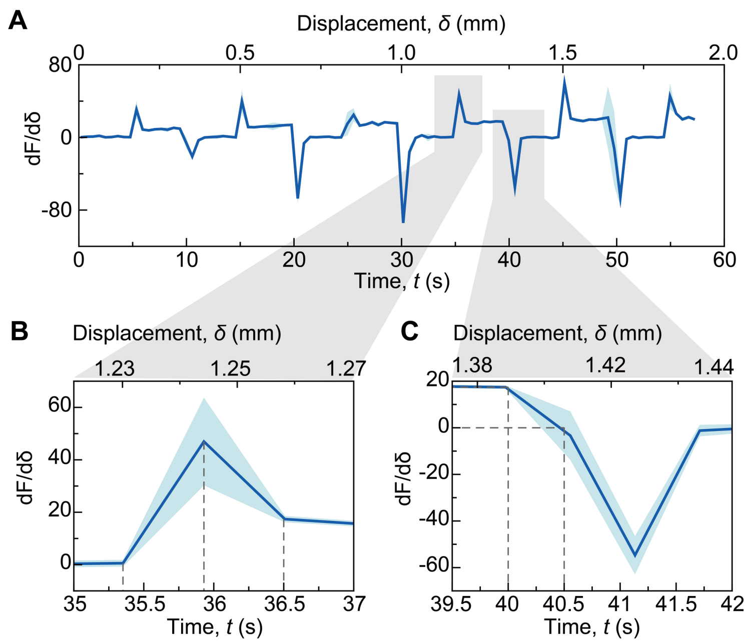

Fig. S13. Dynamic stiffness modulation during mid-test jamming and unjamming. (A) Real-time modulation of stiffness. **(B)** Zoomed-in view of stiffness increase during jamming. **(C)** Zoomed-in view of stiffness reduction during unjamming. Solid lines: mean response (*n* = 5); shaded areas: ± 1 SD.

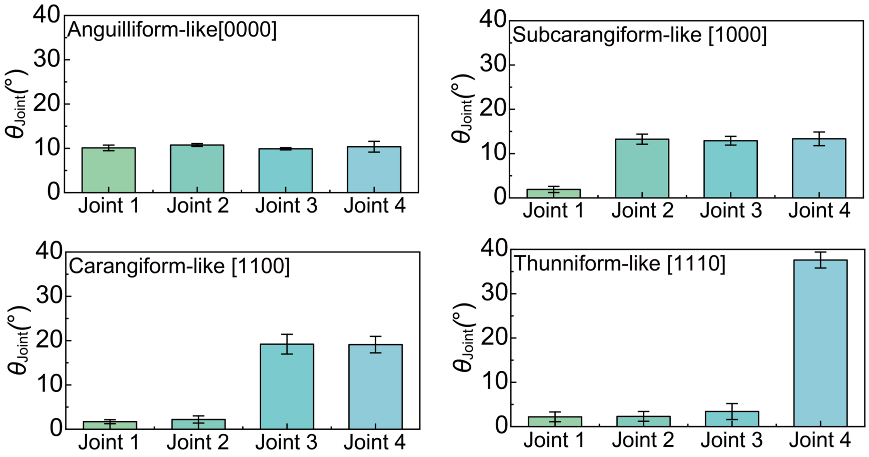

Fig. S14. Joint bending angle θ_{Joint} in different swimming gaits, highlighting mode-specific tail configurations (*n* = 3). All error bars represent ±1 SD.

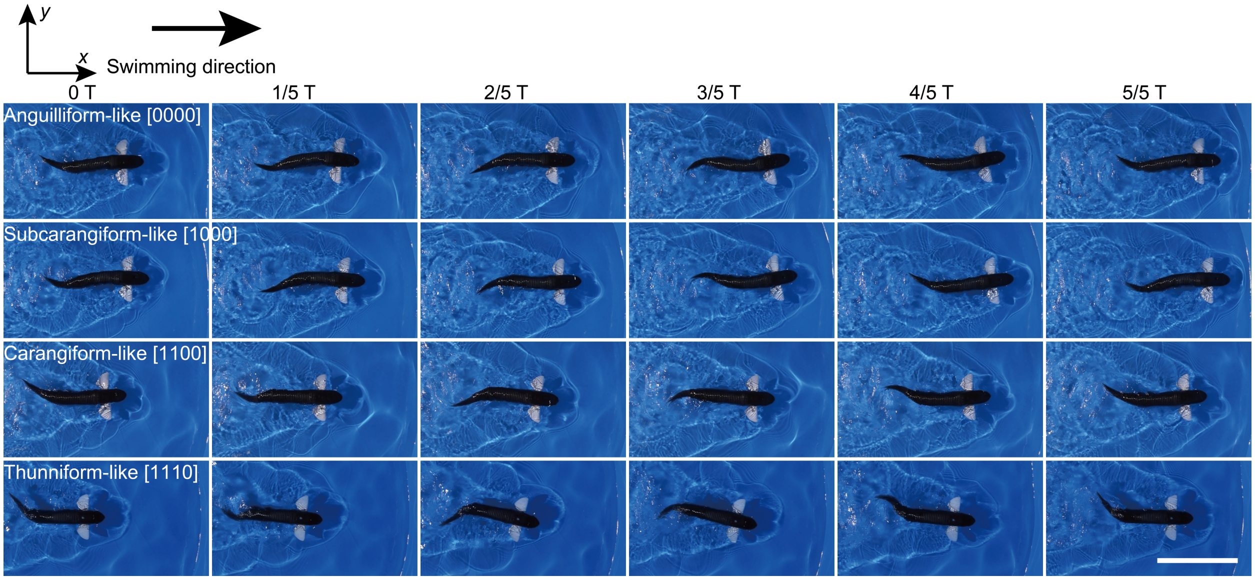

Fig. S15. Snapshots of the robotic fish demonstrating four swimming modes at a swimming frequency of 2 Hz (Movie S4). Scale bar, 50 cm.

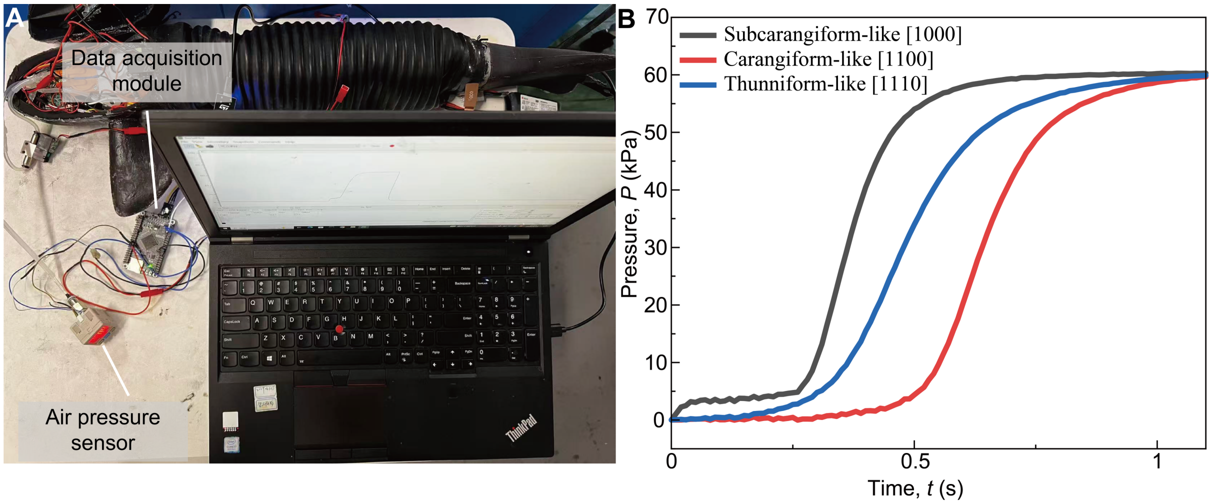

Fig. S16. Measurement of time for switching swimming modal. (A) Experimental setup with an external absolute air-pressure sensor on the vacuum manifold and a data-acquisition module recording $P(t)$ together with valve commands. Tests were performed with the robot held at the neutral midline. **(B)** Pressure traces during jamming for three locking patterns [1000], [1100], [1110]. Stiffness-change time is defined as the interval from the valve command to the moment the manifold pressure reaches the operating setpoint of 60 kPa. Time increases with the number of joints evacuated - [1000] evacuates one joint and is fastest, [1100] two joints, [1110] three joints - and all cases reach the setpoint within about 1 s.

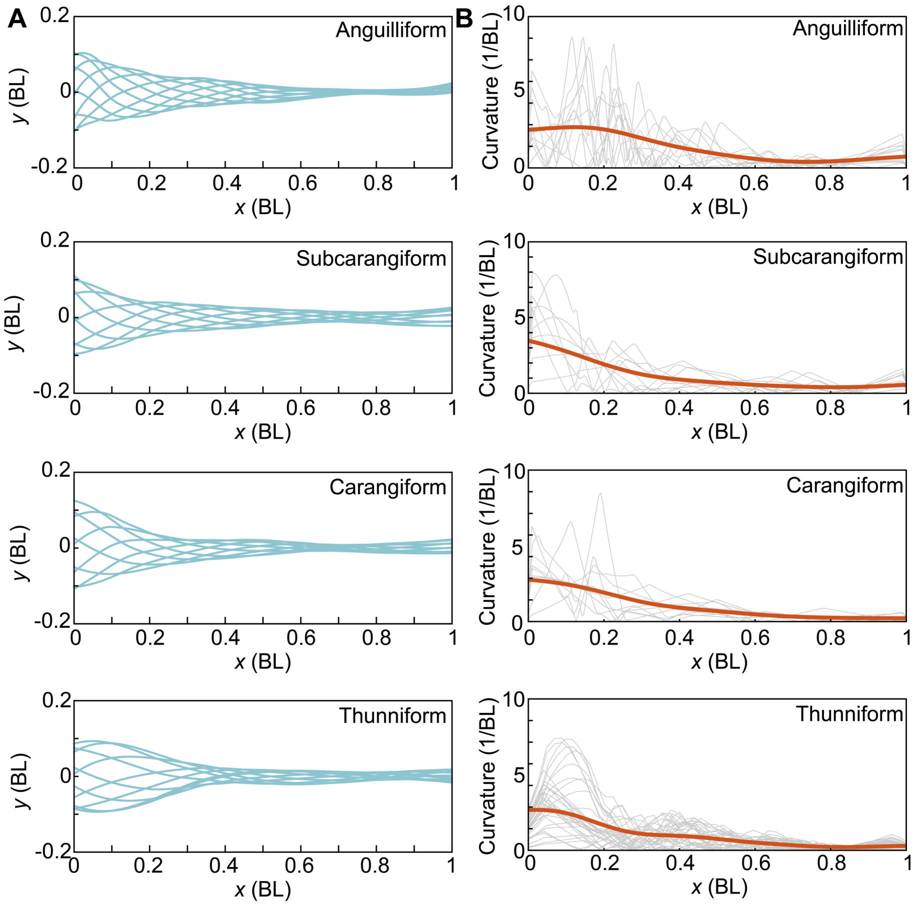

Fig. S17. Body waves of four biological fish species. (A) Body wave profiles of the four biological fish, captured at 10 equally spaced time intervals during a single tail-beat cycle. Data sourced from Reference (*70*). **(B)** Body midline curvature along the fish length; red lines indicate the mean curvature.

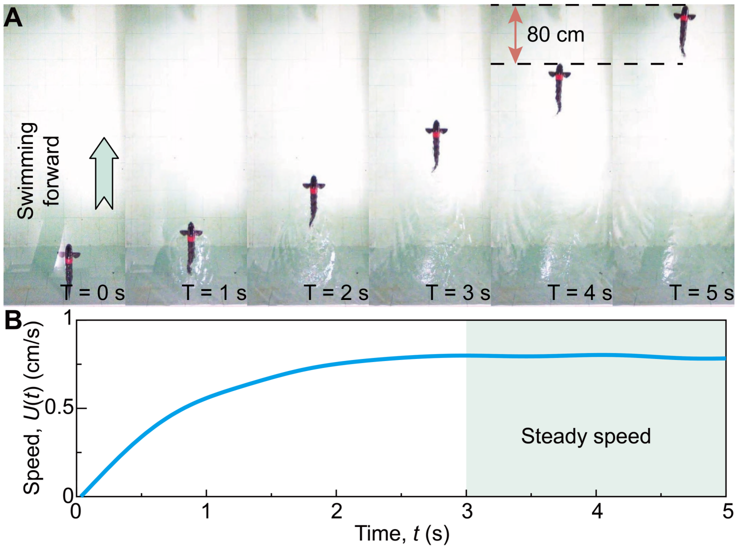

Fig. S18. Forward swimming of robotic fish. (A) Snapshots of a forward swimming process with a 5 Hz driving frequency and thunniform-like [1110] mode (Movie S5 and Movie S6). **(B)** Evolution of the velocity of forward swimming $v(t)$ as a function of time during the swimming process shown in A.

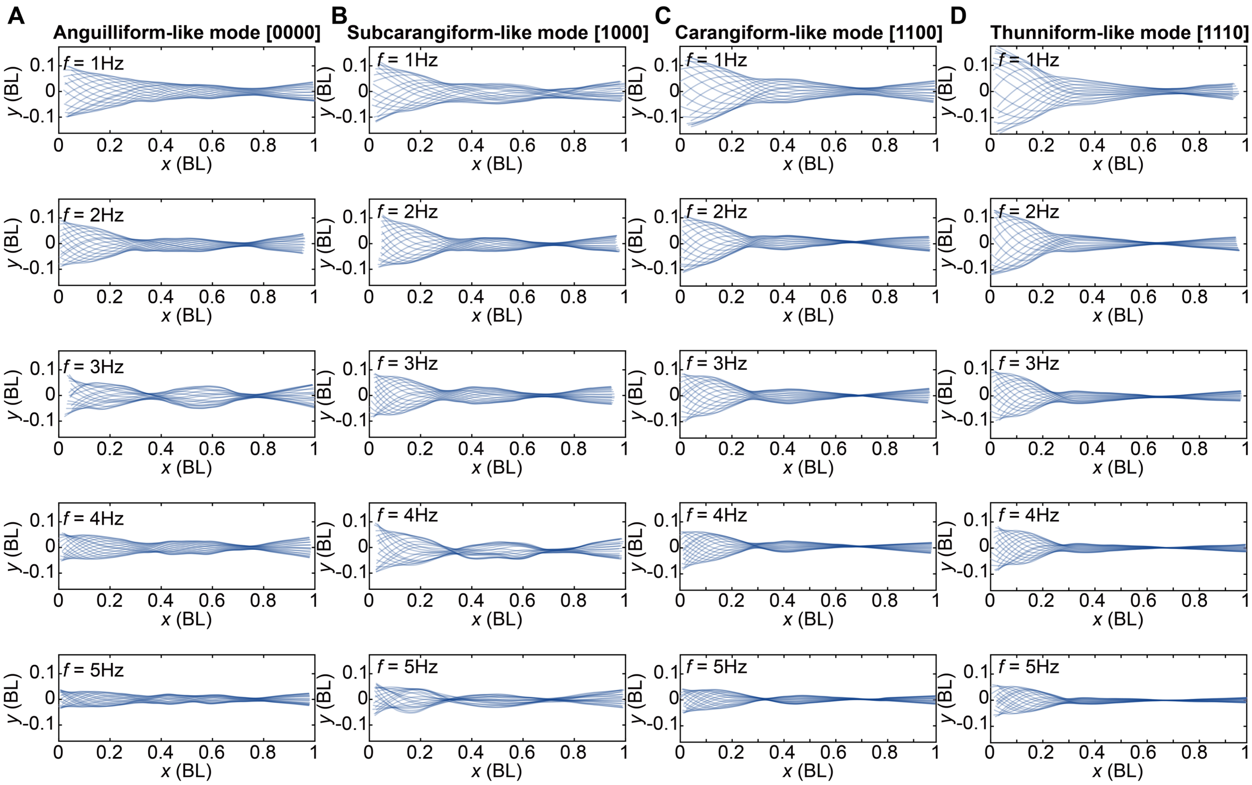

Fig. S19. Body waves of the four swimming modes. (A) Anguilliform-like mode [0000]. **(B)** Subcarangiform-like mode [1000]. **(C)** Carangiform-like mode [1100]. **(D)** Thunniform-like mode [1110].

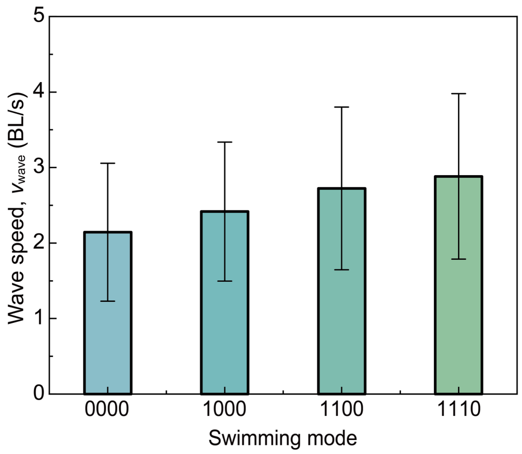

Fig. S20. Average wave speed v_{wave} of the four swimming modes (n = 5). Error bars represent ±1 SD.

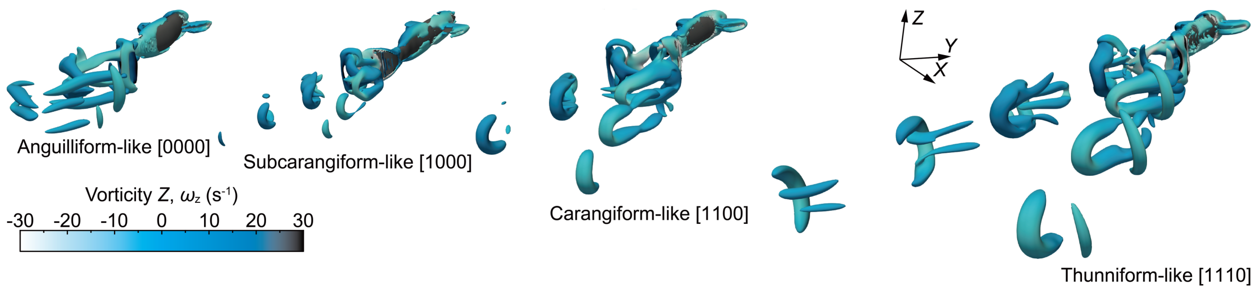

Fig. S21. Three-dimensional isosurfaces of spanwise vorticity ω_z over one cycle, colored by ω_z (s^{-1}), shown for anguilliform-like [0000], subcarangiform-like [1000], carangiform-like [1100], and thunniform-like [1110]. Wakes become progressively more axial with increasing posterior locking (Q = 15).

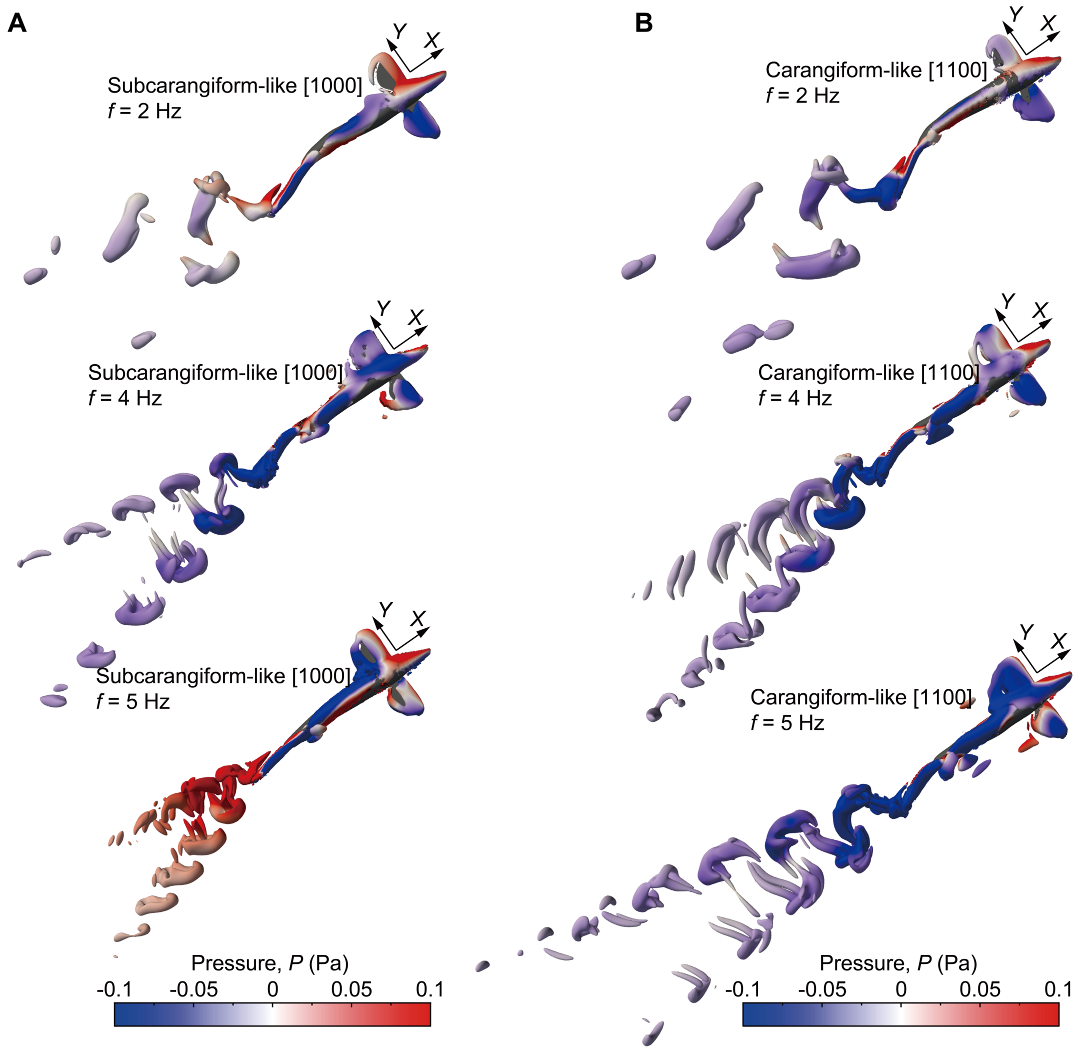

Fig. S22. Q-criterion isosurfaces (*Q* = 15) colored by pressure *P* (Pa) for two swimming gaits: (A) subcarangiform-like [1000] and (B) carangiform-like [1100]. From top to bottom: *f* = 2, 4, 5 Hz.

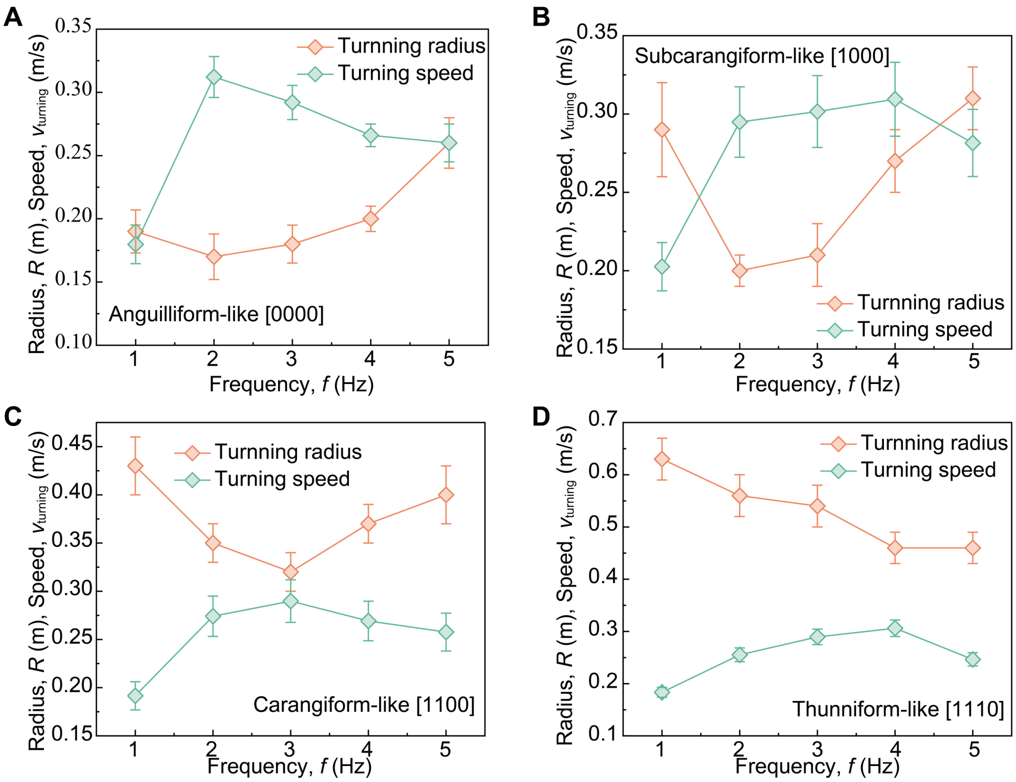

Fig. S23. Turning radius *R* and speed v_{turning} across four gaits. (A–D) Turning radius *R* (orange diamonds, m) and turning speed v_{turning} (green markers, m/s) versus driving frequency *f* for the four modes: anguilliform-like [0000], subcarangiform-like [1000], carangiform-like [1100], and thunniform-like [1110]. Points show mean ± s.e.m. Within each mode, *R* and v_{turning} often vary inversely with *f* ($n = 3$). All error bars represent ±1 SD.

Supplementary Movies

Movie S1. Stiffness modulation of the bioinspired jamming layer muscle (BJLM)

Movie S2. Locking and unlocking of a tensegrity joint

Movie S3. Tail undulation under four joint-locking modes

Movie S4. Demonstration of four swimming gaits

Movie S5. Speed comparison of the four swimming gaits

Movie S6. Swimming speeds of the four gaits at different frequencies

Movie S7. Wake vortex structures across the four swimming gaits

Movie S8. Turning radius under different swimming modes

Movie S9. Real-time online switching between swimming gaits during locomotion

Movie S10. Swimming in constrained environments via dynamic mode switching

Movie S11. Adaptive gait switching for environmental navigation in the wild
